# Improved Sparse Bayesian Off-Grid Direction-of-Arrival Estimation Under Mixed Noise

**DOI:** 10.3390/s26196130

**Published:** 2026-09-27

**Authors:** Jinyi Tian, Xuhu Wang, Yifan Jia, Dazhi Zhang, Yongtao Wang, Yujun Hou

**Affiliations:** 1 School of Information Management, Embodied AI & Robot Research Institute, Qingdao University of Technology, Qingdao 266520, China; tianjinyi@stu.qut.edu.cn (J.T.); jiayifan@stu.qut.edu.cn (Y.J.); zdzhah@163.com (D.Z.); wyyyt1234@163.com (Y.W.); 2 School of Marine Science and Technology, Northwestern Polytechnical University, Xi’an 710129, China; houyujun2024@mail.nwpu.edu.cn

**Keywords:** direction-of-arrival (DOA), mixed noise, sparse Bayesian learning, off-grid estimation, space-alternating, adaptive grid refinement

## Abstract

To improve the robustness and computational efficiency of underwater direction-of-arrival (DOA) estimation in complex marine noise environments, this study proposes an improved sparse Bayesian off-grid DOA estimation method under mixed noise. Within a hierarchical Bayesian framework, temporal precision variables for non-Gaussian noise and spatial precision variables for nonuniform noise are introduced and jointly estimated to adaptively reduce the weights of impulsive snapshots and noise-contaminated array elements. A space-alternating variational inference scheme transforms the joint estimation of signal and noise parameters into low-dimensional analytical updates, thereby reducing the computational burden associated with high-dimensional posterior covariance matrix inversion. In addition, adaptive grid refinement locally densifies high-confidence spectral-peak regions, while a first-order Taylor expansion is employed for continuous off-grid compensation. Simulation results show that the proposed method achieves more stable spatial spectrum reconstruction under mixed-noise and grid-mismatch conditions, providing a favorable balance between estimation accuracy and computational complexity. Results from the SWellEx-96 sea-trial data further demonstrate its ability to stably track target bearings under practical array geometry and unknown oceanic interference. These results verify the effectiveness and applicability of the proposed method in both mixed-noise simulations and real shallow-water data.

## 1. Introduction

With the growing demand for ocean exploration, underwater communications, and sonar localization, direction-of-arrival (DOA) estimation has become an important research topic in array signal processing. DOA estimation aims to determine the incident directions of one or more sources from multichannel observations acquired by an underwater sensor array. Classical subspace-based methods, such as Multiple Signal Classification (MUSIC) and Estimation of Signal Parameters via Rotational Invariance Techniques (ESPRIT) [1,2,3], have been widely applied in underwater detection and sonar localization because of their high resolution under ideal conditions. However, these methods generally rely on idealized assumptions, including a sufficient number of snapshots, accurate covariance-matrix estimation, and independent and identically distributed Gaussian white noise. When the number of snapshots is limited, the signal-to-noise ratio (SNR) is low, or the noise statistics deviate from the Gaussian assumption, the orthogonality between the signal and noise subspaces can be degraded. Consequently, spectral peaks may broaden, spurious peaks may become more pronounced, and angular estimation accuracy may deteriorate. These issues are particularly significant in practical underwater acoustic environments, especially in shallow-water scenarios.

In recent years, the development of compressed sensing [4,5] and sparse reconstruction [6,7] has stimulated considerable interest in sparse Bayesian learning (SBL) [8], owing to its robust performance under low-SNR, few-snapshot, and coherent-source conditions. By imposing sparse priors on source signals in the angular domain, SBL reformulates DOA estimation as a probabilistic sparse-reconstruction problem. Compared with conventional convex-optimization and greedy-search methods, SBL can adaptively estimate signal powers and noise parameters within a Bayesian framework and thereby exhibit good parameter adaptability.

Nevertheless, when the true DOAs do not coincide with the predefined discrete grid points, steering-vector mismatch leads to energy leakage in the sparse spatial spectrum and consequently degrades estimation accuracy. To address this issue, Yang et al. proposed the off-grid sparse Bayesian inference (OGSBI) algorithm [9], which introduces a first-order Taylor expansion to approximate continuous angular deviations and effectively alleviates grid-mismatch errors. However, OGSBI strongly depends on the density of the initial grid, and a finer grid results in substantial computational cost. Dai et al. subsequently proposed Root Sparse Bayesian Learning (Root-SBL) [10], which transforms off-grid optimization into a polynomial root-finding problem. This approach achieves high-accuracy off-grid estimation without relying on a dense grid and substantially improves computational efficiency.

Variational Bayesian inference provides a tractable posterior-approximation framework for sparse recovery and has been applied to angle-of-arrival estimation through iterative Bayesian updates [11]. This line of work motivates the variational formulation adopted in this study, while the proposed method further addresses the mixed-noise, local-grid-correlation, and continuous off-grid issues encountered in underwater array observations.

From the broader perspective of sensing and communication systems, robustness may also be pursued at levels other than the received-array-signal level. For example, the semantic-driven integrated multimodal sensing and communication framework in [12] considers the joint representation, transmission, and task-oriented decoding of multimodal sensing information. In contrast, the present work does not address semantic communication or network-level sensing-service orchestration; it estimates source directions directly from underwater array snapshots under mixed noise and steering-vector mismatch.

Similarly, Tang et al. [13] developed a detection-and-mitigation architecture for low-rate DDoS attacks against software-defined-network flow tables. That study concerns traffic-flow monitoring, attack classification, and flow-table protection at the network layer. The robustness considered here is fundamentally different: temporal and spatial reliability variables are inferred from the acoustic array observations themselves to suppress impulsive snapshots and degraded sensors before sparse DOA reconstruction. The added references therefore clarify the boundary between signal-level mixed-noise robustness in array processing and network-layer detection or mitigation mechanisms.

In practical marine environments, particularly in shallow water, array observations are often affected by multiple types of noise. On the one hand, ship machinery noise, biological noise, and anthropogenic noise may introduce strong impulsive interference into some snapshots, resulting in distinctly non-Gaussian and heavy-tailed noise characteristics [14,15]. On the other hand, sensor aging, channel amplitude-phase errors, and local environmental disturbances may cause unequal noise powers across array elements or even sensor failures, thereby producing spatially nonuniform noise [16,17]. To address DOA estimation under non-Gaussian noise, Ma et al. proposed a robust off-grid SBL method [18], in which heavy-tailed noise modeling and joint estimation of off-grid parameters were employed to suppress the influence of non-Gaussian noise on sparse spectrum reconstruction and angle estimation. For DOA estimation under nonuniform noise, Esfandiari et al. proposed a non-iterative subspace-based method [19], which mitigates subspace-estimation bias caused by unequal sensor-noise powers through nonuniform noise covariance modeling and parameter estimation. However, these methods consider only a single noise type. Therefore, how to jointly address multiple noise types and perform off-grid estimation within a unified Bayesian framework remains a key issue to be addressed.

Moreover, robust DOA estimation methods often introduce additional latent variables [20], weight updates, or high-dimensional matrix operations [21], which further increase computational complexity. In particular, when dense angular grids and complex noise models are simultaneously employed for off-grid estimation, posterior covariance matrix inversion becomes the main computational bottleneck of SBL-based methods [22]. Hence, reducing the complexity of Bayesian inference while improving robustness against mixed noise constitutes another key challenge.

Recent robust and off-grid SBL methods should be distinguished not only by whether an off-grid model is used, but also by the noise structure they represent and the posterior approximation on which their computational efficiency relies. VBI for impulsive and nonuniform noise can jointly model these two noise components [14]. Off-grid SBL has also been developed for impulsive-noise settings [18], while off-grid space-alternating SBL reduces the computational cost of off-grid inference [21]. These studies provide important solutions to individual aspects of robust DOA estimation. However, the coupled setting considered in this work requires impulsive snapshots, degraded sensors, coherent neighboring candidate grids, and continuous grid mismatch to be addressed simultaneously.

The methodological novelty of the proposed framework is not the isolated use of mixed-noise weighting, off-grid correction, or space-alternating inference. Rather, it lies in their identifiable and posterior-coupled integration for mixed-noise off-grid DOA estimation. Specifically, the proposed method makes three linked advances. First, it introduces a separable temporal–spatial reliability model in which temporal precision variables suppress impulsive snapshots and constrained spatial precision variables suppress degraded sensors; the unit-geometric-mean constraint removes the intrinsic scale ambiguity between these two precision components. Second, it replaces a fully scalar posterior approximation over closely spaced candidate grids with coherence-selected local blocks that retain within-block posterior covariance. This design directly addresses adjacent-grid correlation without recovering the prohibitive full-grid covariance inversion. Third, it replaces a manually chosen damping schedule for off-grid correction with posterior-regularized offset estimation, in which the regularization strength and offset uncertainty are inferred from the current VBI statistics and the final offset is restricted to its local grid cell.

More importantly, these three advances are not independent add-on modules. The identified temporal and spatial precisions determine the weighted dictionary and residual statistics; these statistics determine the local-block posterior covariance; and the resulting posterior mean, covariance, and residual jointly determine the continuous off-grid offset. Thus, the proposed method forms a closed posterior-information flow from mixed-noise weighting to correlation-aware sparse reconstruction and then to continuous angular correction. The recent related robust or off-grid SBL methods summarized in Table 1 do not provide this same identifiable, correlation-aware, and posterior-regularized integration. The table is therefore included not merely to list differences, but to make the three-level innovation of the proposed framework explicit.

To address the insufficient robustness and high computational complexity of existing off-grid DOA estimation methods in mixed-noise environments, this study proposes an improved fast sparse Bayesian off-grid DOA estimation method under mixed noise. The main contributions are summarized as follows:

- An identifiable hierarchical mixed-noise model is constructed, in which temporal precision variables downweight impulsive snapshots and constrained spatial precision variables downweight degraded sensors. The unit-geometric-mean constraint separates the relative temporal and spatial reliability scales.
- A local-block space-alternating VBI scheme preserves posterior covariance within coherent neighboring candidate grids while avoiding a full-grid covariance inversion. This explicitly balances the approximation error caused by grid correlation against computational complexity.
- An adaptive grid-refinement and continuous compensation strategy is developed, in which high-confidence neighborhoods are refined by bisection and the residual offset is estimated through a posterior-regularized Taylor update restricted to its local grid cell.

Notation: The superscripts ${\left(\cdot \right)}^{-1}$,${\left(\cdot \right)}^{T}$,${\left(\cdot \right)}^{H}$ and ${\left(\cdot \right)}^{*}$ denote the matrix inversion, the transpose, the conjugate transpose, and the complex conjugation, respectively. The operator diag(·) denotes a diagonal matrix with the elements of its argument on the main diagonal; Tr(·) denotes the matrix trace; ${∥\cdot ∥}_{2}$ denotes the $l_{2}$ norm; and Re{·} denotes the real part of a complex quantity. The mathematical symbols employed throughout the manuscript are listed in Table 2.

## 2. Signal Model

The angular search range is uniformly discretized into *N* coarse grid points, which are denoted by $\bar{\theta }={\left[{\bar{\theta }}_{1},{\bar{\theta }}_{2},\cdots ,{\bar{\theta }}_{N}\right]}^{T}$. Here, *N* denotes the number of candidate grid points. Consider a uniform linear array (ULA) composed of *M* underwater acoustic sensors with an inter-element spacing of *d*. The array receives *K* mutually uncorrelated far-field narrowband signals. Let $\theta_{k}$ denote the true DOA of the *k*-th source. The vector of true DOAs is expressed as $\theta ={\left[\theta_{1},\theta_{2},\cdots ,\theta_{K}\right]}^{T}$. At the *t*-th snapshot, the received array signal is modeled as

(1) y(t)=A(θ)s(t)+n(t)

where $y\left(t\right)={\left[y_{1}\left(t\right),y_{2}\left(t\right),\cdots ,y_{M}\left(t\right)\right]}^{T}$ is the received signal vector of the sensor array, and t=1,2,⋯,T is the snapshot index. The matrix $A\left(\theta \right)=[a\left(\theta_{1}\right),a\left(\theta_{2}\right),\cdots ,a\left(\theta_{K}\right)]$ is the array manifold matrix associated with the true source directions. In addition, $s\left(t\right)={\left[s_{1}\left(t\right),s_{2}\left(t\right),\cdots ,s_{K}\left(t\right)\right]}^{T}$ denotes the source signal vector, and n(t) denotes the mixed-noise vector, which includes both non-Gaussian noise and spatially nonuniform noise and is assumed to be independent of the source signals. For the ULA, the steering vector corresponding to the *k*-th source is given by $a\left(\theta_{k}\right)={\left[1,e^{-j\frac{2\pi d}{\lambda }\sin\left(\theta_{k}\right)},\cdots ,e^{-j\frac{2\pi d}{\lambda }\left(M-1\right)\sin\left(\theta_{k}\right)}\right]}^{T}$, where λ denotes the signal wavelength. By collecting *T* snapshots, the array observation model can be written in matrix form as

(2) Y=AS+N

Based on the initial grid, an overcomplete dictionary matrix is constructed as $\bar{A}=\left[a\left({\bar{\theta }}_{1}\right),a\left({\bar{\theta }}_{2}\right),\cdots ,a\left({\bar{\theta }}_{N}\right)\right]$. The DOA estimation problem can then be reformulated as a spatial sparse-reconstruction problem on the coarse grid:

(3) $$Y=\bar{A}X+N$$

where $X\in C^{N\times T}$ is the sparse signal matrix defined on the coarse grid. Since the actual number of sources, *K*, is typically much smaller than the number of candidate grid points, *N*, only a small number of rows in **X** contain significant energy, whereas the powers of the remaining rows approach zero. To distinguish the true array manifold matrix A(θ) from the coarse-grid dictionary matrix $\bar{A}$, the subsequent Bayesian inference is developed on the basis of the sparse model in (3).

## 3. The Proposed Method

### 3.1. Hierarchical Sparse Bayesian Prior Model

Given the signal precision vector $\gamma ={\left[\gamma_{1},\gamma_{2},\cdots ,\gamma_{N}\right]}^{T}$ at all grid points, it is assumed that the snapshot column vectors x(t) of the sparse signal matrix **X** are mutually independent in the temporal domain and follow zero-mean complex Gaussian distributions.

(4) $$p\left(X|\gamma \right)=\prod_{t=1}^{T}CN\left(x\left(t\right)|0,\Gamma^{-1}\right)$$

where Γ=diag(γ) is the signal precision matrix. Since $\Gamma^{-1}$ is the prior covariance matrix of the sparse signal, a larger $\gamma_{n}$ indicates a smaller signal variance corresponding to the *n*-th grid point, and this grid point is more likely to be suppressed.

A Gamma prior is assigned to $\gamma_{n}$:

(5) $$p\left(\gamma \right)=\prod_{n=1}^{N}\mathrm{Gamma}\left(\gamma_{n}|g,h\right)=\prod_{n=1}^{N}\frac{h^{g}}{\Gamma (g)}\gamma_{n}^{g-1}e^{-h\gamma_{n}}$$

where Γ(·) denotes the Gamma function, and *g* and *h* denote the shape and rate parameters, respectively. In practice, to avoid an excessively strong influence of manually specified prior parameters on sparse power estimation, *g* and *h* are set to extremely small values to yield weakly informative priors during inference.

In conventional SBL models, noise is usually assumed to be independent and identically distributed Gaussian white noise; that is, all array elements and all snapshots share the same noise variance. However, in practical marine environments, owing to impulsive interference from anthropogenic and natural noise sources, as well as sensor aging and channel errors, the noise probability model in practical marine environments exhibits spatial nonuniformity and temporally non-Gaussian characteristics.

Therefore, this study introduces a non-Gaussian noise precision vector $z={\left[z_{1},z_{2},\cdots ,z_{T}\right]}^{T}$ and a nonuniform noise precision vector $\beta ={\left[\beta_{1},\beta_{2},\cdots ,\beta_{M}\right]}^{T}$, and constructs the following conditional likelihood function:

(6) $$p\left(Y|X,\beta ,z\right)=\prod_{t=1}^{T}CN\left(y\left(t\right)|\bar{A}x\left(t\right),z_{t}^{-1}B^{-1}\right)$$

where B=diag(β), $\beta_{m}$ controls the weight of the *m*-th array element in the spatial dimension, and $z_{t}$ controls the weight of the *t*-th snapshot in the temporal dimension. When the noise power of an array element is relatively large or the element partially fails, the corresponding $\beta_{m}$ decreases, thereby reducing the contribution of this array element to power spectrum estimation; when a snapshot is contaminated by strong impulsive noise, $z_{t}$ decreases during inference, thereby reducing the contribution of the abnormal snapshot to parameter updating.

Because the likelihood in Equation (6) depends on the temporal and spatial precisions through the product $z_{t}\beta_{m}$, an unconstrained model has a one-dimensional scale ambiguity: multiplying every $\beta_{m}$ by a positive constant and dividing every $z_{t}$ by the same constant leaves the likelihood unchanged. To make the two precision components identifiable, the physical spatial-precision vector is constrained to have unit geometric mean:

$${\left(\prod_{m=1}^{M}\beta_{m}\right)}^{1/M}=1,\;\mathrm{equivalently},\;\frac{1}{M}\sum_{m=1}^{M}\ln\beta_{m}=0.$$

Accordingly, $\beta_{m}$ represents the relative precision of the *m*-th array element with respect to the array-wide geometric-mean precision, whereas $z_{t}$ represents the snapshot-dependent precision on this identified scale. This constraint preserves the multiplicative effective-precision representation $z_{t}\beta_{m}$ used for robust weighting while preventing arbitrary redistribution of scale between the temporal and spatial components.

The resulting model assumes that temporal and spatial reliability are multiplicatively separable; it is intended to model snapshot-wise impulsive contamination together with sensor-wise heteroscedasticity, rather than an arbitrary nonseparable sensor–snapshot noise covariance.

Under the unit-geometric-mean constraint, the spatial precision vector is regularized by the following constrained Gamma prior:

(7) $$p_{B}\left(\beta \right)=Z_{B}^{-1}\prod_{m=1}^{M}\mathrm{Gamma}\left(\beta_{m}|c_{\beta },d_{\beta }\right),\;\beta \in B,\;B=\left\{\beta \in R_{+}^{M}:\sum_{m=1}^{M}\ln\beta_{m}=0\right\}$$

Here, $c_{\beta }$ and $d_{\beta }$ are the shape and rate parameters, B is the identifiable constraint set, and $Z_{B}$ is the normalizing constant on this set. Consequently, β is treated as a constrained variational hyperparameter and is optimized through a constrained coordinate update. Array elements with larger reconstruction residuals receive smaller relative $\beta_{m}$ values and are downweighted in global spatial-spectrum estimation. The density in Equation (7) is defined with respect to the induced (M−1)-dimensional surface measure on B.

To estimate non-Gaussian noise, a Gamma prior controlled by the degrees-of-freedom parameter *ν* is assigned to $z_{t}$:

(8) $$p\left(z\right)=\prod_{t=1}^{T}\mathrm{Gamma}\left(z_{t}|\frac{\nu }{2},\frac{\nu }{2}\right)$$

where *ν* is used to control the tail heaviness of the non-Gaussian noise model. When *ν* is small, the model has stronger robustness against strong impulses and abnormal snapshots.

### 3.2. Fast Space-Alternating Variational Bayesian Inference

Based on the constrained hierarchical Bayesian model, the latent variables are Θ={X,γ,z}, whereas β is a deterministic variational hyperparameter optimized under the unit-geometric-mean constraint. Combined with the observation data **Y**, the constrained joint objective is

(9) $$p_{B}\left(Y,\Theta ;\beta \right)=p\left(Y|X,\beta ,z\right)p\left(X|\gamma \right)p\left(\gamma \right)p_{B}\left(\beta \right)p\left(z\right)$$

Taking the logarithm of the above joint probability distribution and ignoring constant terms unrelated to the variables to be estimated yields

(10) $$\begin{array}{cc} \ln p_{B}\left(Y,\Theta ;\beta \right)= & \ln p(Y|X,\beta ,z)+\ln p(X|\gamma )+\\ & \ln p\left(\gamma \right)+\ln p_{B}\left(\beta \right)+\ln p\left(z\right) \end{array}$$

The observation likelihood term can be further expanded as

(11) $$\begin{array}{cc} \ln p(Y|X,\beta ,z)= & \sum_{t=1}^{T}\left[M\ln z_{t}+\ln\left|B\right|-z_{t}{\left(y\left(t\right)-\bar{A}x\left(t\right)\right)}^{H}B\right.\\ & \left.\left(y\left(t\right)-\bar{A}x\left(t\right)\right)\right]+\mathrm{const},\;B=\mathrm{diag}\left(\beta \right) \end{array}$$

where const is a constant term.

Since the posterior distribution p(Θ∣Y,β) is difficult to solve directly, this study uses variational Bayesian inference to construct an approximate posterior distribution q(Θ) and jointly maximizes a constrained evidence lower bound with respect to *q* and β [23]. According to variational inference theory, the constrained variational objective J(Y;β) can be decomposed into the constrained evidence lower bound (ELBO) and the Kullback–Leibler divergence:

(12) $$\begin{array}{cc} J(Y;\beta )= & \ln p\left(Y|\beta \right)+\ln p_{B}\left(\beta \right)\\ = & L\left(q,\beta \right)+\mathrm{KL}\left(q(\Theta )∥p(\Theta |Y,\beta )\right)\\ = & \int q\left(\Theta \right)\ln\frac{p_{B}\left(Y,\Theta ;\beta \right)}{q(\Theta )}\;d\Theta -\int q\left(\Theta \right)\ln\frac{p(\Theta |Y,\beta )}{q(\Theta )}\;d\Theta \end{array}$$

Here, J(Y;β) is the constrained variational objective, L(q,β) is its evidence lower bound, and $\mathrm{KL}\left(q(\Theta )∥p(\Theta |Y,\beta )\right)$ is the KL divergence, which represents the approximation degree between the approximate posterior distribution q(Θ) and the posterior distribution conditional on the constrained spatial precision. The smaller the KL divergence, the higher the approximation degree.

At the top level, the variational posterior is factorized across the signal, signal-precision, and temporal-precision variable groups, while q(X) is subsequently represented by the structured local-block factorization in Equation (15):

(13) q(Θ)=q(X)q(γ)q(z)

According to variational inference theory, for fixed constrained β, the optimal approximate posterior distribution $q^{*}\left(\Theta_{i}\right)$ of any latent-variable subset $\Theta_{i}\in \Theta$ is obtained by maximizing L(q,β), and its logarithmic form satisfies the following general variational update rule:

(14) $$\ln q^{*}\left(\Theta_{i}\right)={\langle \ln p_{B}\left(Y,\Theta ;\beta \right)\rangle }_{q(\Theta \setminus \Theta_{i})}+\mathrm{const}$$

where ${\langle \cdot \rangle }_{q(\Theta \setminus \Theta_{i})}$ denotes the expectation with respect to all variables in the set Θ except $\Theta_{i}$.

The discrete steering dictionary is generally coherent at adjacent candidate angles. Consequently, the following posterior factorization is used only as a structured variational approximation and does not assert physical posterior independence among adjacent grid coefficients. At each adaptive-grid level, the active grid points are partitioned into disjoint local blocks ${\left\{G_{b}\right\}}_{b=1}^{B}$. Contiguous candidates with weighted dictionary coherence no smaller than a prescribed threshold $\tau_{c}$ are assigned to the same block, subject to a maximum block size $G_{\max}$, whereas isolated candidates are treated as singleton blocks. The weighted coherence between two candidate atoms is $\kappa_{i,j}=\left|{\bar{a}}_{i}^{H}\hat{B}{\bar{a}}_{j}\right|/\sqrt{\left({\bar{a}}_{i}^{H}\hat{B}{\bar{a}}_{i}\right)\left({\bar{a}}_{j}^{H}\hat{B}{\bar{a}}_{j}\right)}$. The block partition is updated after each grid-refinement level using the current weighted dictionary and candidate peak neighborhoods.

(15) $$q\left(X\right)=\prod_{t=1}^{T}\prod_{b=1}^{B}q\left(x_{G_{b},t}\right),\;q\left(x_{G_{b},t}\right)=\mathrm{CN}\left(x_{G_{b},t}|\mu_{G_{b},t},\Sigma_{G_{b},t}\right).$$

Here, $x_{G_{b},t}$ collects the coefficients indexed by $G_{b}$ at snapshot *t*, $\Sigma_{G_{b},t}$ retains their within-block posterior covariance, and a singleton block reduces exactly to the original scalar update. For the *b*-th block, the residual after removing the current block contribution is defined as

(16) $$r_{G_{b},t}=y\left(t\right)-\sum_{c\neq b}{\bar{A}}_{G_{c}}\mu_{G_{c},t}.$$

(1) Updating the Sparse Signal **X**

Conditioned on the current expectations of the other latent variables, completing the square gives a multivariate complex Gaussian update for each local block:

(17) $$q^{*}\left(x_{G_{b},t}\right)=CN\left(x_{G_{b},t}|\mu_{G_{b},t},\Sigma_{G_{b},t}\right).$$

(18) $$\Sigma_{G_{b},t}={\left(\langle z_{t}\rangle {\bar{A}}_{G_{b}}^{H}\hat{B}{\bar{A}}_{G_{b}}+\Gamma_{G_{b}}\right)}^{-1}.$$

(19) $$\mu_{G_{b},t}=\Sigma_{G_{b},t}\langle z_{t}\rangle {\bar{A}}_{G_{b}}^{H}\hat{B}r_{G_{b},t}.$$

where ${\bar{A}}_{G_{b}}$ contains the dictionary columns in $G_{b}$ and $\Gamma_{G_{b}}=\mathrm{diag}\left(\langle \gamma_{G_{b}}\rangle \right)$. Let $\Sigma_{t}^{\mathrm{blk}}=\mathrm{blkdiag}\left(\Sigma_{G_{1},t},\ldots ,\Sigma_{G_{B},t}\right)$ denote the block-diagonal covariance used in the remaining updates. To implement the update efficiently, the global residual is maintained recursively. The superscripts “old” and “new” below distinguish the block mean before and after the update in Equation (19); after each block update, $R^{\mathrm{new}}$ is used as $R^{\mathrm{old}}$ for the next block.

(20) $$R^{\mathrm{old}}=Y-\bar{A}\mu^{\mathrm{old}}.$$

(21) $$R_{G_{b}}=R^{\mathrm{old}}+{\bar{A}}_{G_{b}}\mu_{G_{b},:}^{\mathrm{old}}.$$

(22) $$R^{\mathrm{new}}=R_{G_{b}}-{\bar{A}}_{G_{b}}\mu_{G_{b},:}^{\mathrm{new}}.$$

Thus, the algorithm avoids inversion of the full N×N covariance matrix while preserving posterior correlations inside the selected local blocks.

To quantify the approximation caused by neglecting only inter-block correlations, define the conditional Gaussian precision and its block-diagonal counterpart as

(23) $$\Lambda_{t}=\langle z_{t}\rangle {\bar{A}}^{H}\hat{B}\bar{A}+\Gamma ,\;\Lambda_{\mathrm{blk},t}=\mathrm{blkdiag}\left(\Lambda_{G_{1},G_{1},t},\ldots ,\Lambda_{G_{B},G_{B},t}\right).$$

At a coordinate fixed point, the block-wise mean equals the mean of this conditional Gaussian posterior. Because $q_{t}$ and $p_{t}$ are proper complex Gaussian distributions, their KL divergence does not contain the 1/2 prefactor associated with real Gaussian densities. Therefore, the conditional divergence induced by the block approximation is

(24) $$D_{\mathrm{KL}}\left(q_{t}\;∥\;p_{t}\right)=\log\frac{\det\left(\Lambda_{\mathrm{blk},t}\right)}{\det\left(\Lambda_{t}\right)}=-\log\det\left(I+E_{t}\right),\;E_{t}=\Lambda_{\mathrm{blk},t}^{-1/2}\left(\Lambda_{t}-\Lambda_{\mathrm{blk},t}\right)\Lambda_{\mathrm{blk},t}^{-1/2}.$$

Because $\mathrm{Tr}(E_{t})=0$, if ${∥E_{t}∥}_{2}<1$, this divergence satisfies

(25) $$D_{\mathrm{KL}}\left(q_{t}\;∥\;p_{t}\right)\leq \frac{{∥E_{t}∥}_{F}^{2}}{2\left(1-{∥E_{t}∥}_{2}\right)}.$$

This is a conditional-posterior bound for fixed $\hat{B}$, $\langle z_{t}\rangle$, and 〈γ〉; it does not claim a bound for the complete hierarchical posterior. The bound decreases as the remaining inter-block precision coupling decreases, which motivates grouping strongly correlated neighboring grids in the same local block.

(2) Updating the Nonuniform Noise Precision

Under the unit-geometric-mean constraint, β is updated by maximizing the constrained expected log joint rather than by using an unconstrained factorized Gamma posterior:

(26) $$\hat{\beta }=\underset{\beta_{m}>0,\;\prod_{m=1}^{M}\beta_{m}=1}{\arg\max}\;\sum_{m=1}^{M}\left[\left(c_{\beta }+T-1\right)\ln\beta_{m}-{\tilde{d}}_{m}\beta_{m}\right]$$

The expected residual coefficient is

(27) $$\begin{array}{cc} {\tilde{d}}_{m}= & d_{\beta }+\sum_{t=1}^{T}\langle z_{t}\rangle \left({\left|y_{m}\left(t\right)-{\bar{a}}_{m,:}\mu_{:,t}\right|}^{2}+{\bar{a}}_{m,:}\Sigma_{t}^{\mathrm{blk}}{\bar{a}}_{m,:}^{H}\right) \end{array}$$

Introducing a Lagrange multiplier for $\sum_{m}\ln\beta_{m}=0$ and solving the stationarity condition yield

(28) $${\hat{\beta }}_{m}=\frac{{\left(\prod_{j=1}^{M}{\tilde{d}}_{j}\right)}^{1/M}}{{\tilde{d}}_{m}},\;m=1,\ldots ,M,\;\hat{B}=\mathrm{diag}\left(\hat{\beta }\right).$$

where ${\bar{a}}_{m,:}$ denotes the *m*-th row of the discrete dictionary matrix $\bar{A}$, $\mu_{:,t}$ denotes the posterior mean vector of all grid signals at the *t*-th snapshot, and ${\bar{a}}_{m,:}\mu_{:,t}$ denotes the reconstructed signal of the *m*-th array element at the *t*-th snapshot.

Equation (28) is the constrained variational coordinate update and guarantees ${\left(\prod_{m=1}^{M}{\hat{\beta }}_{m}\right)}^{1/M}=1$ at every iteration. Hence, a malfunctioning array element with a larger reconstruction residual has a smaller relative spatial precision and is downweighted, without introducing an arbitrary scale trade-off with $z_{t}$.

(3) Updating the Non-Gaussian Noise Precision

The approximate posterior distribution of $z_{t}$ is

(29) $$q^{*}\left(z_{t}\right)=\mathrm{Gamma}\left(z_{t}|{\tilde{a}}_{t},{\tilde{b}}_{t}\right)$$

The parameter update formulas are

(30) $${\tilde{a}}_{t}=\frac{\nu }{2}+M$$

(31) $$\begin{array}{cc} {\tilde{b}}_{t}= & \frac{\nu }{2}+{\left(y\left(t\right)-\bar{A}\mu_{:,t}\right)}^{H}\hat{B}\left(y\left(t\right)-\bar{A}\mu_{:,t}\right)\\ & +\mathrm{Tr}\left(\hat{B}\bar{A}\Sigma_{t}^{\mathrm{blk}}{\bar{A}}^{H}\right) \end{array}$$

Its expectation is $\langle z_{t}\rangle ={\tilde{a}}_{t}/{\tilde{b}}_{t}$. When the *t*-th snapshot contains strong impulsive noise, a relatively large weighted residual is produced, causing ${\tilde{b}}_{t}$ to increase and $\langle z_{t}\rangle$ to decrease, thereby achieving adaptive suppression of the abnormal snapshot. In each iteration, this temporal-precision update is evaluated using the geometrically normalized $\hat{B}$; therefore, the inferred temporal and spatial precision components remain separately interpretable on the identified scale.

(4) Updating the Signal Precision

The approximate posterior distribution of the signal precision $\gamma_{n}$ is determined by the posterior second moment of the signal component at the *n*-th grid point:

(32) $$q^{*}\left(\gamma_{n}\right)=\mathrm{Gamma}\left(\gamma_{n}|{\tilde{g}}_{n},{\tilde{h}}_{n}\right)$$

The parameter update formulas are

(33) $${\tilde{g}}_{n}=g+T$$

(34) $${\tilde{h}}_{n}=h+\sum_{t=1}^{T}\left(|\mu_{n,t}{|}^{2}+{\left[\Sigma_{t}^{\mathrm{blk}}\right]}_{n,n}\right)$$

Its expectation is $\langle \gamma_{n}\rangle ={\tilde{g}}_{n}/{\tilde{h}}_{n}$. When the *n*-th grid point does not contain a true signal, the corresponding posterior second moment $|\mu_{n,t}{|}^{2}+{\left[\Sigma_{t}^{\mathrm{blk}}\right]}_{n,n}$ is relatively small, causing $\langle \gamma_{n}\rangle$ to increase and the corresponding prior variance ${\langle \gamma_{n}\rangle }^{-1}$ to decrease, thereby achieving sparse suppression of the signal component at this grid point.

Since $\gamma_{n}$ denotes the signal precision, a larger value indicates a smaller signal power at the corresponding grid point. To facilitate spatial spectrum construction, spectral peak detection, and adaptive grid refinement, this study further defines the equivalent signal power at the *n*-th grid point as $\rho_{n}=1/\langle \gamma_{n}\rangle$. Subsequent spatial spectrum estimation is developed based on ρ.

Considering that directly calculating the global ELBO incurs a large computational cost, this study uses the relative change rate of the equivalent power spectrum as the convergence criterion for variational Bayesian inference iterations:

(35) $$\frac{{∥\rho^{\left(i\right)}-\rho^{\left(i-1\right)}∥}_{2}}{{∥\rho^{\left(i-1\right)}∥}_{2}}<\varepsilon$$

where *ε* is the convergence tolerance. When the relative change rate of the power spectrum *ρ* satisfies the above expression or the maximum number of iterations $T_{\max}$ is reached, the iteration is terminated.

#### Convergence of the Fixed-Grid VBI Stage

The convergence statement below concerns one inner VBI stage with a fixed dictionary $\bar{A}$ and a fixed local-block partition ${\left\{G_{b}\right\}}_{b=1}^{B}$. It does not treat grid insertion, outer candidate pruning, or Taylor compensation as iterations of one unchanged variational objective. Let $\xi =\left(q(X),q(\gamma ),q(z),\beta \right)$ denote the variational state, where β∈B. For the fixed-grid stage, assume that the variational factors have finite second moments, the Gamma hyperparameters are proper, and the positive numerical precision bounds used in the implementation define a bounded feasible set. Under these conditions, the constrained ELBO L(ξ) is finite, continuous, and upper bounded.

In one VBI iteration, the local-block Gaussian updates in Equations (17)–(22) maximize L with respect to one block factor at a time while the remaining factors are fixed. The constrained spatial-precision update in Equation (28), the temporal-precision update in Equations (29)–(31), and the signal-precision update in Equations (32)–(34) are respectively the exact maximizers of their corresponding constrained coordinate subproblems. Therefore, if $\xi^{\left(i,0\right)}$ is the state at the beginning of the *i*-th fixed-grid iteration, then $\xi^{\left(i,1\right)}$, $\xi^{\left(i,2\right)}$, $\xi^{\left(i,3\right)}$, and $\xi^{\left(i,4\right)}$ denote the states after the block-signal, spatial-precision, temporal-precision, and signal-precision updates, respectively, with $\xi^{\left(i+1,0\right)}=\xi^{\left(i,4\right)}$.

(36) $$L\left(\xi^{\left(i,0\right)}\right)\leq L\left(\xi^{\left(i,1\right)}\right)\leq L\left(\xi^{\left(i,2\right)}\right)\leq L\left(\xi^{\left(i,3\right)}\right)\leq L\left(\xi^{\left(i,4\right)}\right)=L\left(\xi^{\left(i+1,0\right)}\right).$$

Hence, the ELBO sequence generated by the fixed-grid VBI stage is nondecreasing and converges to a finite limit. Because every coordinate update satisfies the first-order optimality condition of its block subproblem, every accumulation point of the iterates is a coordinatewise stationary point of the constrained ELBO:

(37) $$0\in {\left.\partial_{\xi }\left[-L\left(\xi \right)+\iota_{B}\left(\beta \right)\right]\right|}_{\xi =\xi^{★}},$$

where $\iota_{B}$ is the indicator function of the unit-geometric-mean constraint set. This result establishes convergence to a stationary point of the structured variational problem, rather than global optimality. The relative-spectrum criterion in Equation (35) is used as a practical termination test for this convergent inner stage. The outer adaptive-grid procedure performs only a finite number of refinement levels, and the subsequent off-grid step performs a finite number of local coordinate updates; consequently, the complete algorithm terminates after a finite number of outer operations, while no monotonic-ELBO claim is made across changes of dictionary dimension.

### 3.3. Adaptive Grid Refinement Based on the Bisection Method

Off-grid error mainly originates from the incomplete match between the true incident angle and the predefined discrete grid. Although directly using a global dense grid can reduce grid-mismatch error, it significantly increases the dictionary dimension and the complexity of Bayesian inference. To balance estimation accuracy and computational complexity, this study adopts an adaptive grid refinement strategy based on bisection. A relatively coarse grid is used at the initial stage. After the initial spatial spectrum is obtained through variational Bayesian inference, local densification is performed around high-confidence spectral-peak neighborhoods.

Based on the equivalent signal power $\rho_{n}$, the normalized spatial spectrum is defined as

(38) $$P_{n}=\frac{\rho_{n}}{\max_{i}\rho_{i}}$$

where $\rho_{i}$ denotes the equivalent signal power of the *i*-th discrete grid point, i=1,2,…,N, and $\max_{i}\rho_{i}$ denotes the maximum equivalent signal power among all candidate grid points.

If the *n*-th grid point simultaneously satisfies the local peak condition and exceeds the threshold, it is regarded as a candidate target grid point:

(39) $$\begin{array}{c} P_{n}>\tau^{\left(r\right)},\;P_{n}>P_{n-1},\;P_{n}>P_{n+1},\;n=2,\ldots ,N-1 \end{array}$$

where $\tau^{\left(r\right)}$ is the spectral-peak detection threshold adaptively determined at refinement level *r*.

For the candidate grid ${\bar{\theta }}_{n}$, the powers of its left and right neighboring grids are compared, and a midpoint grid is inserted on the side with the larger power:

(40) $$\theta_{\mathrm{new}}=\frac{{\bar{\theta }}_{n}+{\bar{\theta }}_{\mathrm{neighbor}}}{2}$$

where ${\bar{\theta }}_{\mathrm{neighbor}}$ is the grid point with the larger normalized power among the left and right neighboring grids of ${\bar{\theta }}_{n}$. To avoid erroneously deleting potential target regions at an early stage, the initial base grid is always retained during the refinement process; for subsequently added non-base grids, if their powers remain below the adaptively determined pruning threshold or they are no longer located in candidate spectral-peak neighborhoods, they can be deleted from the current dictionary to control the dictionary size. If the minimum spacing between the current neighboring grids is no greater than the predefined tolerance $\bar{\Delta }$, grid refinement is terminated:

(41) $$\min_{n}\left|{\bar{\theta }}_{n+1}-{\bar{\theta }}_{n}\right|\leq \bar{\Delta }$$

To intuitively illustrate the adaptive grid refinement mechanism of the proposed method, Figure 1 shows the grid evolution process of the proposed algorithm in a GMM mixed-noise environment. The experimental conditions are consistent with those of the experiment shown in Figure 2 below.

In Figure 1, the red circles denote the candidate grids, the green triangles denote high-confidence candidate peaks, and the blue dashed vertical lines mark the true DOAs.

In Figure 2, the grey dashed vertical lines mark the true DOAs.

At the initial stage, the algorithm performs sparse spectrum reconstruction using a coarse grid covering the entire direction search interval. Then, according to the comparison of the powers in the left and right neighborhoods of the candidate peaks, a midpoint grid is inserted on the side with the stronger power, thereby achieving bisection refinement of potential source regions. At the same time, the initial base grid is always retained to avoid erroneously deleting weak sources or target regions affected by mixed noise at an early stage; the newly added non-base grids are dynamically retained or pruned according to the current power spectrum, thereby controlling the dictionary size.

As shown in the figure, grid refinement resources are mainly concentrated near the two true directions, and the local grid spacing is sequentially refined from $8^{\circ }$ to $4^{\circ }$, $2^{\circ }$, and $1^{\circ }$. When the local grid spacing reaches the predefined tolerance, the algorithm records the grid at which refinement is terminated and passes the corresponding candidate region to the subsequent first-order Taylor off-grid compensation for continuous direction correction. This process avoids global dense-grid searching and reduces discrete-grid mismatch error while maintaining a relatively low computational burden.

### 3.4. Continuous Off-Grid Compensation Based on First-Order Taylor Expansion

After adaptive grid refinement is terminated, the target direction may still lie between adjacent points on the final refined grid. To further improve estimation accuracy, continuous off-grid compensation is performed on the final refined grid. Let the true angle be $\theta_{k}={\bar{\theta }}_{k}+\delta_{k}$. A first-order Taylor expansion is performed on the steering vector of the final discrete grid ${\bar{\theta }}_{k}$:

(42) $$\begin{array}{c} a\left(\theta_{k}\right)\approx a\left({\bar{\theta }}_{k}\right)+b\left({\bar{\theta }}_{k}\right)\delta_{k} \end{array}$$

All angles in this subsection, including $\theta_{k}$, ${\bar{\theta }}_{k}$, and $\delta_{k}$, are expressed in degrees. Accordingly, $b\left({\bar{\theta }}_{k}\right)={\left.\frac{\partial a(\theta )}{\partial \theta_{\deg}}\right|}_{\theta ={\bar{\theta }}_{k}}$ is the steering-vector derivative with respect to degrees, and $\delta_{k}$ denotes the continuous offset in degrees. Substituting the above expression into the weighted residual term and retaining only the quadratic terms related to $\delta_{k}$ yields the following objective function:

(43) Fk(δk)=Pkδk2−2Re{Qk}δk+const

The quadratic coefficient $P_{k}$ and the complex coefficient of the linear term $Q_{k}$ are explicitly constructed from the posterior statistics inferred by the current VBI:

(44) $$P_{k}=\sum_{t=1}^{T}\langle z_{t}\rangle \left(|\mu_{k,t}{|}^{2}+{\left[\Sigma_{t}^{\mathrm{blk}}\right]}_{k,k}\right)b{\left({\bar{\theta }}_{k}\right)}^{H}\hat{B}b\left({\bar{\theta }}_{k}\right)$$

(45) $$\begin{array}{cc} Q_{k}= & \sum_{t=1}^{T}\langle z_{t}\rangle [\mu_{k,t}^{*}b{\left({\bar{\theta }}_{k}\right)}^{H}\hat{B}\left(r_{k,t}-a\left({\bar{\theta }}_{k}\right)\mu_{k,t}\right)\\ \; & -b{\left({\bar{\theta }}_{k}\right)}^{H}\hat{B}{\bar{A}}_{G(k)}{\left[\Sigma_{t}^{\mathrm{blk}}\right]}_{G(k),k}]. \end{array}$$

where $r_{k,t}=y\left(t\right)-\sum_{i\neq k}\bar{a}\left({\bar{\theta }}_{i}\right)\mu_{i,t}$ is the residual vector after removing the *k*-th candidate component. Here, G(k) denotes the local block containing the *k*-th candidate, and ${\left[\Sigma_{t}^{\mathrm{blk}}\right]}_{G(k),k}$ is the corresponding within-block covariance column. The second term in Equation (45) retains the full within-block covariance contribution, including cross-covariances with the other coefficients in the local block. If the adaptive offset prior is omitted, taking the first-order derivative of Equation (43) and setting it to zero yields the following unregularized stationary point, which is used only as a reference for the posterior-regularized update below:

(46) δ˜k=Re{Qk}Pk

Rather than selecting a fixed damping factor, the off-grid offset is assigned a zero-mean Gaussian prior with an adaptive precision $\alpha_{k}$: $p\left(\delta_{k}|\alpha_{k}\right)=N\left(\delta_{k}|0,\alpha_{k}^{-1}\right)$. The final point estimate is restricted to its local grid cell, where $\delta_{\max,k}=\frac{1}{2}\min_{j\neq k}\left|{\bar{\theta }}_{k}-{\bar{\theta }}_{j}\right|$ is half the distance to the nearest remaining grid point; for an interior point, this is equivalently $\frac{1}{2}\min\left\{|{\bar{\theta }}_{k}-{\bar{\theta }}_{k-1}\left|,\right|{\bar{\theta }}_{k+1}-{\bar{\theta }}_{k}|\right\}$. A Gamma hyperprior $p\left(\alpha_{k}\right)=\mathrm{Gamma}\left(\alpha_{k}|a_{\delta },b_{\delta ,k}\right)$ enables the regularization strength to be inferred from the current posterior statistics.

(47) vδ,k=Pk+〈αk〉−1,μδ,k=Re{Qk}Pk+〈αk〉.

The adaptive precision is updated by the variational Gamma posterior

(48) $$q^{*}\left(\alpha_{k}\right)=\mathrm{Gamma}\left(\alpha_{k}|a_{\delta }+\frac{1}{2},\;b_{\delta ,k}+\frac{1}{2}\left(\mu_{\delta ,k}^{2}+v_{\delta ,k}\right)\right),\;\langle \alpha_{k}\rangle =\frac{a_{\delta }+\frac{1}{2}}{b_{\delta ,k}+\frac{1}{2}\left(\mu_{\delta ,k}^{2}+v_{\delta ,k}\right)}.$$

Equations (47) and (48) are alternated for a small fixed number of local coordinate updates. The final offset is the projected posterior mean:

(49) $${\hat{\delta }}_{k}={\mathrm{Proj}}_{\left[-\delta_{\max,k},\;\delta_{\max,k}\right]}\left(\mu_{\delta ,k}\right),\;\lambda_{k}=\frac{\langle \alpha_{k}\rangle }{\max(P_{k},\varepsilon )}.$$

Thus, for $P_{k}\geq \varepsilon$, the original denominator can be written as $P_{k}+\langle \alpha_{k}\rangle =P_{k}+\lambda_{k}\left|P_{k}\right|$, but $\lambda_{k}$ is now determined by the current posterior curvature, residual-driven linear term, and offset uncertainty rather than by a manually selected damping schedule.

Finally, the target direction estimate is obtained as ${\hat{\theta }}_{k}={\bar{\theta }}_{k}+{\hat{\delta }}_{k}$.

### 3.5. Computational Complexity Analysis

Let $G_{\max}=\max_{b}\left|G_{b}\right|$ be the largest local-block size. Let $N_{d}$ be the number of fixed dense grid points, $\bar{N}$ be the maximum number of grid points during the adaptive refinement process, ${\bar{N}}_{a}$ be the maximum number of effective grid points updated in each iteration of the proposed method, and *R* be the maximum number of grid refinement levels. $I_{O}$, $I_{R}$, $I_{A}$, and $I_{P}$ denote the numbers of iterations of OGSBI, RSBL, AGRSBL, and the proposed method, respectively; $\bar{Q}$ is the average number of search points in each candidate spectral-peak neighborhood of AGRSBL; and $I_{\delta }$ is the number of off-grid compensation iterations.

The main computational procedures of FLOM-MUSIC [24] include fractional lower-order moment matrix construction, subspace decomposition, and full-angle scanning. Its computational complexity is

(50) $$C_{\mathrm{FLOM}-\mathrm{MUSIC}}=O\left[M^{2}T+M^{3}+M\left(M-K\right)N_{d}\right]$$

In each iteration, OGSBI updates the off-grid dictionary, posterior mean, posterior covariance, signal hyperparameters, and noise precision. Since its posterior covariance update involves high-dimensional matrix operations on a dense grid, its computational complexity can be expressed as

(51) $$\begin{array}{c} C_{\mathrm{OGSBI}}=O\left\{I_{O}\left[MN_{d}^{2}+M^{2}N_{d}+MN_{d}T+M^{3}\right]\right\} \end{array}$$

RSBL [25] characterizes normal observations and abnormal noise through a mixture model and alternately estimates sparse signals and abnormal noise within a variational Bayesian framework. However, its signal posterior update still involves high-dimensional posterior covariance matrix operations on a dense angular grid. Therefore, its computational complexity is approximately

(52) $$\begin{array}{c} C_{\mathrm{RSBL}}=O\left\{I_{R}\left[MN_{d}^{2}+M^{2}N_{d}+MN_{d}T+M^{3}\right]\right\} \end{array}$$

AGRSBL [26] reduces the requirement for global dense searching through layer-by-layer grid refinement, but array covariance updates are still performed at each level, and local searches are executed in candidate spectral-peak neighborhoods. Therefore, its computational complexity can be written as

(53) $$\begin{array}{c} C_{\mathrm{AGRSBL}}=O\left\{RI_{A}\left(M^{3}+M^{2}\bar{N}+M\bar{N}T\right)+RK\bar{Q}\left(M^{2}+MT\right)\right\} \end{array}$$

The proposed method updates the posterior statistics, array-element precision, and snapshot precision only on effective grids. The local-block update adds inversions of matrices with order at most $G_{\max}$, rather than an inversion of the full ${\bar{N}}_{a}\times {\bar{N}}_{a}$ covariance matrix. Its computational complexity is

(54) $$C_{\mathrm{Proposed}}=O\left\{RI_{P}T\left[M{\bar{N}}_{a}+\sum_{b=1}^{B}{\left|G_{b}\right|}^{3}\right]+I_{\delta }KMT\right\}.$$

The first term corresponds to block-structured variational updates under multi-level adaptive grids, and the second term corresponds to first-order Taylor off-grid compensation for a finite number of candidate sources. Because $\sum_{b=1}^{B}{\left|G_{b}\right|}^{3}\leq G_{\max}^{2}{\bar{N}}_{a}$ and $G_{\max}\ll {\bar{N}}_{a}$ by design, the method avoids the global high-dimensional covariance inversion required by dense-grid SBL.

## 4. Simulation Results and Analysis

To verify the off-grid DOA estimation performance of the proposed method under mixed-noise environments, this study constructs Gaussian mixture model noise and symmetric *α*-stable noise environments, respectively, and further superimposes array-element nonuniform noise and random array-element failures in both types of non-Gaussian noise. The proposed method is compared with the Off-Grid Sparse Bayesian Inference (OGSBI) algorithm, the Fractional Lower Order Moment Multiple Signal Classification (FLOM-MUSIC) algorithm, the Robust Sparse Bayesian Learning (RSBL) algorithm, and the Adaptive Grid Refinement Sparse Bayesian Learning (AGRSBL) algorithm. The simulation experiments evaluate algorithm performance from four aspects: spatial spectrum reconstruction, root mean square error, estimation success probability, and running time.

### 4.1. Simulation Setup

The simulation uses a uniform linear array composed of 16 array elements, with a sound speed of 1500 m/s. The experiments uniformly use a center frequency of 750 Hz and an inter-element spacing of 1 m, corresponding to half a wavelength. The true incident angles of the two far-field narrowband sources are set to $-13.14^{\circ }$ and $20.27^{\circ }$, respectively, and the signal amplitudes follow a zero-mean unit-variance complex Gaussian distribution. Unless otherwise stated, the ranges of SNR or GSNR are both −10dB∼10dB with an interval of 5 dB.

To comprehensively verify the robustness of the proposed method under different types of non-Gaussian noise, this study uses GMM noise [27] and S*α*S noise [28] to construct simulation experiments, respectively. GMM noise describes background noise and occasional impulsive noise through a mixture of Gaussian components with different variances. Its variance is finite, making it suitable for simulating environments with intermittent abnormal interference; S*α*S noise has more pronounced heavy-tailed characteristics and does not have a finite variance, making it suitable for describing strong impulsive noise. The use of these two noise models with different statistical characteristics can avoid experimental conclusions relying on a single non-Gaussian noise assumption and can more comprehensively verify the robustness and applicability of the proposed method in marine noise environments.

GMM noise is modeled as

(55) $$n_{m,t}=\left(1-q_{m,t}\right)n_{1,m,t}+q_{m,t}n_{2,m,t}$$

where $q_{m,t}∼\mathrm{Bernoulli}\left(\varpi \right)$, *ϖ* denotes the impulsive probability; the background noise and impulsive noise satisfy

(56) $$n_{1,m,t}∼CN\left(0,\sigma_{1}^{2}\right)$$

(57) $$n_{2,m,t}∼CN\left(0,\sigma_{2}^{2}\right)$$

In the experiment, the impulsive probability is set to ϖ=10%, the impulsive-noise power amplification factor is set to κ=100, and

(58) $$\sigma_{1}^{2}=\frac{10^{-\mathrm{SNR}/10}}{(1-\varpi )+\varpi \kappa }$$

(59) $$\sigma_{2}^{2}=\kappa \sigma_{1}^{2}$$

To simulate nonuniform noise, in each independent experiment, the noise variance coefficients of all array elements are randomly generated from the interval [0.5,2.5]. Then, two degraded array elements are randomly selected, their received signals are set to zero, and their corresponding noise variance coefficients are amplified by a factor of 10. The above nonuniform-noise coefficients and degraded-array-element settings are applied to both the GMM and S*α*S noise experiments.

The characteristic function of S*α*S noise is

(60) $$\varphi \left(\omega \right)=e^{-{\left|\omega \right|}^{\alpha }}$$

where *ω* is the independent variable of the characteristic function, and *α* is the characteristic exponent, with a range of 0<α≤2. Moreover, the smaller *α* is, the more pronounced its impulsive behavior becomes. Unless otherwise stated, α=1.4 is used; the characteristic-exponent experiment below varies *α* to examine the effect of different impulsiveness levels. Because S*α*S noise does not possess a finite second-order moment when α<2, the generalized signal-to-noise ratio (GSNR) is used instead of the conventional SNR to characterize the relative strength between the target signal and the S*α*S noise. A larger GSNR corresponds to a lower effective noise level.

All experiments were completed in MATLAB R2022a. The computing platform is configured with an Intel Core i7-14650HX processor, 16 GB memory, and the Windows 11 operating system.

The proposed method uses an initial $8^{\circ }$ grid over $\left[-90^{\circ },90^{\circ }\right]$ and locally refines candidate-peak neighborhoods by bisection until the grid-refinement tolerance $\bar{\Delta }=1^{\circ }$ is reached. The weighted-coherence threshold for local-block construction is $\tau_{c}=0.95$, with a maximum block size of $G_{\max}=3$. The spectral-peak detection threshold and pruning threshold are adaptively determined from the current normalized spectrum to retain effective candidate regions while controlling the dictionary size. The estimated source number equals the number of retained spectral peaks after screening by minimum peak separation and relative peak strength.

The posterior-regularized off-grid update uses $a_{\delta }=10^{-3}$ and $b_{\delta ,k}=10^{-3}\delta_{\max,k}^{2}$, with three local coordinate updates. Rather than being manually fixed, the equivalent damping coefficient is inferred from the current posterior statistics. The maximum offset $\delta_{\max,k}$ is restricted to half the distance from candidate *k* to its nearest remaining grid point. The fixed-grid VBI stage uses the relative equivalent-power change in Equation (35) as its stopping criterion, with $\varepsilon =10^{-5}$ and a maximum of 3000 iterations. Signal-related hyperparameters are initialized from the Bartlett spectrum, spatial precision is initialized from the sample covariance, and temporal precision is initialized to one.

For the comparison methods, OGSBI uses a 181-point uniform grid, a tolerance of $10^{-4}$, and a maximum of 2000 iterations. AGRSBL uses a $12^{\circ }$ initial grid, a stopping threshold of $10^{-3}$, and a maximum of 500 iterations. RSBL uses a 361-point uniform grid, Gamma hyperparameters of 0.01, a tolerance of $10^{-4}$, and a maximum of 500 iterations. FLOM-MUSIC uses a 361-point search grid, a fractional lower-order-moment parameter of p=1.2, and a subspace dimension of K=2.

### 4.2. Evaluation Metrics

This study uses the root mean square error to measure angular estimation error. To avoid label switching among multiple sources, one-to-one matching should first be performed according to the minimum total angular error. Unless otherwise stated, the main performance comparison experiments use $L_{\mathrm{MC}}=300$ Monte Carlo trials; the GMM impulsive-probability and key-parameter sensitivity analyses use 100 trials per setting. In the *l*-th Monte Carlo experiment, the true direction of arrival is $\theta_{k}$, and the estimated angle matched with it is ${\hat{\theta }}_{\pi_{l}\left(k\right)}^{\left(l\right)}$. Here $l=1,2,\cdots ,L_{\mathrm{MC}}$ is the Monte Carlo experiment index, k=1,2,⋯,K is the true direction-of-arrival index, and $\pi_{l}\left(k\right)$ is the one-to-one matching relationship determined by the minimum total angular error criterion in the *l*-th experiment.

The root mean square error is defined as

(61) $$\mathrm{RMSE}=\sqrt{\frac{1}{L_{\mathrm{MC}}K}\sum_{l=1}^{L_{\mathrm{MC}}}\sum_{k=1}^{K}{\left({\hat{\theta }}_{\pi_{l}\left(k\right)}^{\left(l\right)}-\theta_{k}\right)}^{2}}$$

The unit of RMSE is degrees ${(}^{\circ })$. This metric simultaneously reflects the overall angular estimation errors of different sources and different Monte Carlo experiments. A smaller value indicates higher estimation accuracy of the algorithm.

This study uses the source-wise success probability to measure the ability of the algorithm to correctly estimate source directions. In each trial, the estimated directions are first matched one-to-one with the true directions. When the estimated source number equals the true source number, each matched source whose angular error does not exceed the predefined tolerance $\Delta_{\theta }$ is counted as a successful directional estimate; otherwise, all sources in that trial are counted as unsuccessful. In this study, $\Delta_{\theta }=1^{\circ }$. The source-wise success probability is defined as

(62) $$\begin{array}{c} \mathrm{Success}\;\mathrm{Probability}=\frac{1}{L_{\mathrm{MC}}K}\sum_{l=1}^{L_{\mathrm{MC}}}\sum_{k=1}^{K}I\left[{\hat{K}}^{\left(l\right)}=K,\;\left|{\hat{\theta }}_{\pi_{l}\left(k\right)}^{\left(l\right)}-\theta_{k}\right|\leq \Delta_{\theta }\right] \end{array}$$

where ${\hat{K}}^{\left(l\right)}$ is the number of sources obtained in the *l*-th experiment, and I[·] is the indicator function, which takes the value of 1 when the condition inside the brackets is satisfied and 0 otherwise. Since K=2 in the reported experiments, $L_{\mathrm{MC}}=300$ trials produce 600 source-level indicators at each SNR or GSNR point.

The running time of the algorithm is represented by the average over multiple independent runs:

(63) $${\bar{T}}_{\mathrm{run}}=\frac{1}{L_{\mathrm{time}}}\sum_{l=1}^{L_{\mathrm{time}}}T_{\mathrm{run}}^{\left(l\right)}$$

where $L_{\mathrm{time}}$ denotes the number of repetitions for the running-time test, $T_{\mathrm{run}}^{\left(l\right)}$ denotes the running time of the algorithm estimation stage in the *l*-th test, and ${\bar{T}}_{\mathrm{run}}$ denotes the average running time, with the unit of seconds. The timing scope includes only the estimation process of the algorithm and does not include observation-data generation, result saving, or plotting. All algorithms are run under the same hardware platform, software environment, and experimental parameters.

### 4.3. Estimation Performance Under Mixed Noise

Figure 2 shows the spatial spectrum estimation results of the five algorithms under GMM mixed noise at an SNR of 0 dB. This noise environment simultaneously includes non-Gaussian noise interference, nonuniform noise, and random array-element failures.

As shown in Figure 2, the proposed method can form clear spectral peaks near the two true directions, and the estimated locations are relatively close to the true DOAs, thereby effectively suppressing the interference caused by mixed noise; the other four methods can only estimate the approximate directions of the incident sources, with relatively low estimation accuracy.

This study uses the root mean square error to quantify the estimation errors of the five algorithms. Figure 3 shows the comparison results under GMM mixed noise, with the number of snapshots set to 100.

To further examine robustness to variations in the impulsive-noise environment, Figure 4 reports the RMSEs of the five methods as the GMM impulsive probability *q* varies from 0 to 0.30. The number of snapshots, array configuration, nonuniform-noise setting, and degraded-array setting are kept the same as those in Figure 3.

For this test, the SNR is fixed at 0 dB and the impulsive-to-background noise power ratio is set to κ=100. As shown in Figure 4, the proposed method maintains a stable sub-degree RMSE throughout the tested range, indicating that its estimation accuracy is not strongly affected by moderate changes in the occurrence probability of impulsive samples. OGSBI has substantially larger errors over the entire range, whereas AGRSBL exhibits a pronounced increase in error when *q* reaches 0.10. These results further demonstrate the stability of the proposed method under GMM mixed noise with different impulsive-occurrence levels.

Figure 5 shows that the RMSEs generally decreased as the SNR or GSNR increased. With 100 snapshots, the updated proposed-method curves were obtained from 300 Monte Carlo trials at each noise level. The proposed method achieved an RMSE of 0.6746° at −10 dB under GMM mixed noise. At −10 dB under S*α*S mixed noise, it achieved 6.9641°. Both values were lower than those of the four comparison methods. Under S*α*S mixed noise, the RMSE fell to 0.4463° at −5 dB and remained below 0.26° from 0 to 10 dB. The gaps among the methods narrowed under GMM mixed noise as the SNR increased, whereas the proposed method retained the lowest RMSE across the tested GSNR range under S*α*S mixed noise.

To further evaluate robustness under different levels of impulsiveness, the characteristic exponent *α* of the S*α*S noise was varied while the GSNR was fixed at 0 dB and the number of snapshots was set to 100. The tested values were α=2.0, 1.8, 1.6, 1.4, and 1.3, where a smaller *α* indicates stronger impulsiveness and heavier tails. For every value of *α*, 100 independent Monte Carlo trials were conducted for each method.

As shown in Figure 6, the RMSEs of all five methods increase as *α* decreases from 2.0 to 1.3, but the extent of degradation differs considerably. The RMSE of the proposed method increases only slightly, from 0.2063° to 0.2490°, and remains below 0.25° throughout the tested range. By contrast, the four comparison methods exhibit substantially larger RMSE increases as the S*α*S noise becomes more impulsive. These results show that, under the specified 0 dB GSNR and 100-snapshot condition, the proposed method is comparatively insensitive to changes in the characteristic exponent and retains stable DOA estimation accuracy over the tested range.

Figure 7 and Figure 8 show the source-wise DOA estimation success probabilities of the five algorithms under GMM and S*α*S mixed noise, respectively. The numbers of snapshots are set to 100 for the GMM experiment and 200 for the S*α*S experiment. At each SNR or GSNR point, 300 independent Monte Carlo trials are performed. In each trial, the two true source directions are matched one-to-one with the estimates and evaluated separately. A source-level result is regarded as successful only when the estimated source number is correct and the absolute error of the matched DOA does not exceed 1°. Consequently, each reported success probability is calculated from 600 source-level decisions, obtained by evaluating two sources in each of the 300 trials, rather than from 300 binary trial-level outcomes.

As shown in the figures, the success probabilities of the comparison methods generally increase as the SNR or GSNR increases. At −10 dB, the proposed method achieves source-wise success probabilities of 88.00% under GMM mixed noise and 54.83% under S*α*S mixed noise, both substantially higher than those of the four comparison methods. Under GMM mixed noise, its success probability increases from 98.67% at −5 dB to 99.83% at 10 dB. Under S*α*S mixed noise, it increases from 79.17% at −5 dB to 99.00% at 10 dB, indicating robust performance as the GSNR improves. The modest fluctuations at individual SNR or GSNR points result from the finite Monte Carlo sample and the randomly varying locations and amplitudes of strong impulsive samples, without changing the overall conclusion.

Figure 9 compares the average running times of the five methods under GMM mixed noise, with the number of snapshots uniformly set to 100. The timing scope includes only the algorithm estimation process and does not include data generation, result saving, or plotting.

As shown in Figure 9, the average running times of FLOM-MUSIC, RSBL, and AGRSBL are approximately 0.003 s, 0.02 s, and 0.1 s, respectively; that of the proposed method is approximately 0.29 s, whereas OGSBI has the longest running time. Although the proposed method is not the fastest among the five methods, it significantly reduces the computational burden of traditional off-grid algorithms and achieves higher estimation accuracy and success probability within a relatively short running time. Therefore, the proposed method achieves a relatively good balance among mixed-noise robustness, off-grid estimation accuracy, and computational efficiency.

### 4.4. Sensitivity Analysis of Key Parameters

To verify that the performance of the proposed method does not rely on a narrowly tuned parameter configuration, Figure 10 evaluates its sensitivity to the grid-refinement tolerance $\bar{\Delta }$, candidate-peak prominence threshold *T*, local-block coherence threshold $\tau_{c}$, and off-grid prior scale $\rho_{\delta }$. Only the proposed method is considered. The SNR is fixed at 0 dB, with q=0.10 and κ=100; all remaining settings are identical to those used in Figure 3. Each parameter value is evaluated over 100 independent Monte Carlo trials, while the other parameters retain their default values.

The RMSE remains below 0.57° when $\bar{\Delta }$ varies from 0.25° to $1^{\circ }$, whereas overly coarse refinement degrades the estimation accuracy. Therefore, $\bar{\Delta }=1^{\circ }$ is adopted as an accuracy–complexity compromise. The RMSE is nearly unchanged for T=4–8 dB and varies only slightly as $\rho_{\delta }$ changes from 0.1 to 10, indicating that T=6 dB and $\rho_{\delta }=1$ lie in stable operating regions. For $\tau_{c}$, the RMSE remains within 0.40–0.50° over the practical interval from 0.90 to 0.995. The default value $\tau_{c}=0.95$ is retained because it provides a stable local-block partition consistent with the weighted-coherence criterion. Overall, the adopted parameter configuration was empirically validated and selected by jointly considering estimation accuracy, stability over practical operating ranges, computational cost, and the theoretical constraints of the proposed framework.

Figure 10a–d correspond to $\bar{\Delta }$, *T*, $\tau_{c}$, and $\rho_{\delta }$, respectively; red inverted triangles plot the RMSE values and black dashed vertical lines mark the default settings.

## 5. Sea-Trial Data Processing and Analysis

### 5.1. Sea-Trial Description

This study is further validated using the SWellEx-96 sea trial. SWellEx-96 is a typical shallow-water acoustic experiment conducted approximately 12 km offshore of Point Loma, San Diego, California, USA, from 10 to 18 May 1996. As shown in Figure 11, the experiment deployed a vertical line array, a tilted line array, and two bottom-mounted horizontal line arrays, namely HLA North and HLA South, and included multiple towed-source events. This study selects the measured HLA North data from Event S59 to conduct sea-trial validation of the proposed method. Event S59 lasted approximately 65 min. During the experiment, the source vessel R/V Sproul traveled at approximately 5 kn near the 180 m isobath, while a relatively strong moving interference vessel was present in the experimental area. Therefore, these data contain complex factors such as real shallow-water propagation, multipath effects, and nonstationary interference [29].

HLA North was deployed on the seabed at a water depth of approximately 213 m, with an array aperture of approximately 240 m and an array-axis direction of 34.5° east of north. As shown in Figure 12, the array contains 32 elements, and 27 valid elements were retained after abnormal channels were removed. The data sampling frequency is 3276.8 Hz. This study constructs the actual array manifold according to the corrected array-element coordinates officially provided, in order to avoid simply idealizing the sea-trial array as an equally spaced uniform linear array. In Event S59, R/V Sproul simultaneously towed the deep source J-15 and the shallow source J-13. The towing depth of J-15 was approximately 54 m, and it could transmit multiple groups of single-frequency signals in the range of 49–400 Hz. This study selects the 148 Hz component in the high-source-level single-frequency group of source J-15 as the target signal, and the source level of this frequency component is approximately 158 dB. The GPS trajectory of the source vessel was converted into incident angles relative to the axis direction of the HLA North array and was used as an external reference for the DOA estimation results.

In Figure 12, the numbers label the original hydrophone indices, and the blue markers identify the 27 retained valid elements.

To ensure the continuity of the target narrowband component and the GPS reference trajectory, this study selects data from 9–10 min after the start of the S59 recording for analysis and uses the same data segment and array parameters to conduct a fair comparison of the five algorithms. It should be noted that the noise distribution, array-element response errors, and interference composition in the measured SWellEx-96 data are all unknown. Therefore, this section does not directly equate them with the predefined non-Gaussian noise and nonuniform noise models. This sea-trial experiment is mainly used to examine the generalization ability of the proposed method under real ocean propagation environments and unknown interference conditions; the suppression performance for specific mixed-noise models is verified by the preceding controlled simulation experiments.

### 5.2. Data Processing and Analysis

This study analyzes the data from 9–10 min after the start of the S59 event recording. First, the DC components of the data from the 27 valid array elements are removed, and framing and complex demodulation are performed using a 1024-point Hann window to extract the narrowband component around 148 Hz. Considering the slight Doppler frequency shift caused by the motion of the towed source, the analysis frequency is corrected according to the line-spectrum peak within each data window. Subsequently, the array manifold is constructed based on the measured two-dimensional coordinates of the array elements to retain the geometric characteristics of the actual deployment configuration of HLA North.

To ensure fairness, the proposed method and the four comparison methods use the same array-element data, array geometry, analysis period, and snapshot settings. The bearing-time experiments uniformly use 100 snapshots and construct 41 overlapping data windows within 9–10 min. Because the definitions and amplitude scales of the spatial spectra differ among the algorithms, the spatial spectrum of each method is normalized by the maximum value within each time window, and the displayed dynamic range is uniformly set to −15–0 dB.

In Figure 13, the cyan dashed line represents the reference bearing calculated from the GPS trajectory, and the solid line with circular markers represents the DOA estimation trajectory of each method. All five methods can form continuous spectral peaks near the target bearing; however, the estimation trajectory of the proposed method is overall more consistent with the GPS reference trajectory and maintains good continuity throughout the entire analysis period. It should be noted that each subplot uses independent peak normalization. Therefore, the color intensity only reflects the relative spectral distribution within the corresponding method and cannot be directly used to compare the absolute background power of different methods.

To further evaluate the sea-trial DOA estimation performance of the methods, the number of snapshots is set to 25, 50, 75, 100, 125, and 150, while the total observation duration is kept consistent under different snapshot numbers. Using the GPS reference bearing as the benchmark, the root mean square errors of the five methods within the same time interval are calculated, and the results are shown in Figure 14.

As shown in Figure 14, the proposed method achieves the lowest RMSE under all snapshot settings. Its RMSE remains between 1.29° and 1.54°, with an average value of approximately 1.37°; the average RMSE of FLOM-MUSIC, which has the closest performance, is approximately 1.67°, and the proposed method achieves a relative reduction of approximately 17.6%. Combining the bearing-time records and RMSE results, the proposed method demonstrates good bearing-tracking accuracy and stability under actual array geometry, shallow-water propagation, and unknown interference conditions. Because the noise statistical characteristics of the sea-trial data are unknown, these results are mainly used to verify the general applicability of the method in actual ocean environments.

To examine whether the snapshot-dependent result generalizes across sea-trial events, an additional experiment was conducted using the HLA North data from the independent SWellEx-96 S5 event. The 148 Hz component was evaluated over nine temporal windows from 15.5 to 16.5 min. For 25, 50, and 75 snapshots, 100 subsets were randomly sampled without replacement from a fixed 100-snapshot observation aperture in each temporal window; the 100-snapshot setting used the complete aperture. The five methods used the same data subsets, array geometry, and GPS-derived bearing reference in every trial. The resulting RMSEs are shown in Figure 15.

As shown in Figure 15, the RMSEs of all methods generally decrease as the number of snapshots increases. The proposed method achieves the lowest RMSE at every tested snapshot count, decreasing from 1.70° at 25 snapshots to 1.30° at 100 snapshots. This result from the independent S5 event is consistent with the S59 result in Figure 14, indicating that the proposed method maintains favorable snapshot efficiency and bearing-estimation accuracy under different measured shallow-water conditions.

To further assess robustness to temporal variations in the measured environment, the five methods were evaluated over six separated time segments of the SWellEx-96 S5 event, namely 8.0–9.1, 15.5–16.6, 23.0–24.1, 34.2–35.4, 38.0–39.1, and 45.5–46.6 min after the start of recording. For each segment, 100 independent snapshot-resampling trials were conducted by randomly selecting 100 snapshots from a fixed 200-snapshot candidate set. The five methods used the same selected snapshots, array geometry, target frequency, and GPS-derived bearing reference in every trial. The resulting RMSEs are shown in Figure 16.

As shown in Figure 16, the proposed method achieves the lowest RMSE in all six time segments. Although the absolute errors of all methods vary with the propagation condition and interference level of each segment, the proposed method maintains consistently lower estimation errors, demonstrating stable bearing-estimation performance under temporally varying shallow-water environments. Together with the snapshot-number experiments in Figure 14 and Figure 15, this result indicates that the proposed method is robust not only to changes in the available observation snapshots but also to temporal variations in the real sea-trial environment.

## 6. Conclusions

This study presents an improved sparse Bayesian off-grid method for underwater DOA estimation in the presence of impulsive snapshots, sensor-dependent nonuniform noise, and grid mismatch. The main contribution is an integrated posterior-inference framework comprising three closely coupled components. Constrained temporal and spatial precision variables provide identifiable reliability weighting for abnormal snapshots and degraded sensors; coherence-selected local-block variational inference preserves the posterior correlation between neighboring candidate grids without requiring full-grid covariance inversion; and adaptive grid refinement combined with posterior-regularized Taylor compensation provides continuous DOA correction within the corresponding local grid cells. Together, these components address mixed-noise suppression, neighboring-grid correlation, off-grid estimation, and computational tractability within a unified framework.

Under the tested two-source simulation conditions, the proposed method produced well-localized spatial spectra and showed favorable RMSE and source-wise success-probability performance under both GMM and S*α*S mixed noise. At −10 dB, its RMSEs were $0.6746^{\circ }$ under GMM mixed noise and $6.9641^{\circ }$ under S*α*S mixed noise, while the corresponding source-wise success probabilities were 88.00% and 54.83%, respectively. The advantage was particularly evident under severe low-SNR or low-GSNR conditions, whereas the success probabilities of several methods became comparable as the noise level decreased. The GMM impulsive-probability, S*α*S characteristic-exponent, and parameter-sensitivity experiments further showed that the proposed method remained stable over the tested practical ranges. Complexity analysis and running-time measurements confirmed that the local-block update avoids global high-dimensional covariance inversion and requires substantially less running time than OGSBI. Although it is not the fastest of the five methods, it provides a favorable balance among estimation accuracy, mixed-noise robustness, and computational cost.

The SWellEx-96 results provide complementary evidence beyond the controlled noise simulations. Using the measured HLA North geometry and GPS-derived bearing as references, the proposed method maintained continuous target-bearing estimates in the S59 event and achieved an average RMSE of approximately 1.37° across the tested snapshot settings, representing a 17.6% reduction relative to the closest comparison method. In the independent S5 event, it also achieved the lowest RMSE at every tested snapshot count and across six separated time segments. These results support the applicability of the proposed method to temporally varying shallow-water observations with unknown interference. Nevertheless, the current validation is limited to narrowband processing at a selected frequency, a specific array configuration, and selected segments from two SWellEx-96 events. Future work will examine broadband and multi-source scenarios, stronger array-manifold mismatch, and real-time implementations on embedded or field-deployable processing platforms.

## Figures and Tables

**Figure 1:**
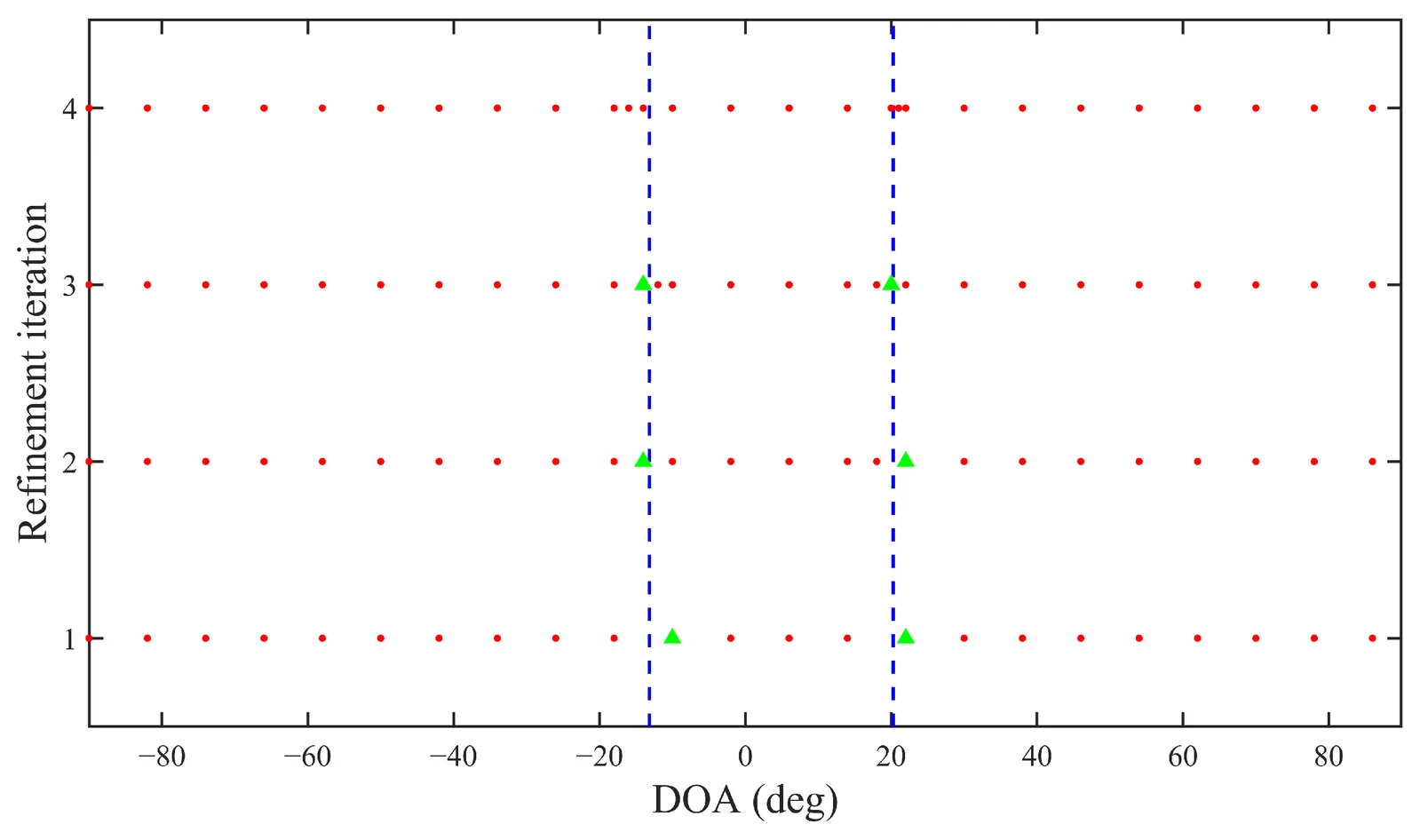
Adaptive Grid Refinement Process under GMM Mixed Noise.

**Figure 2:**
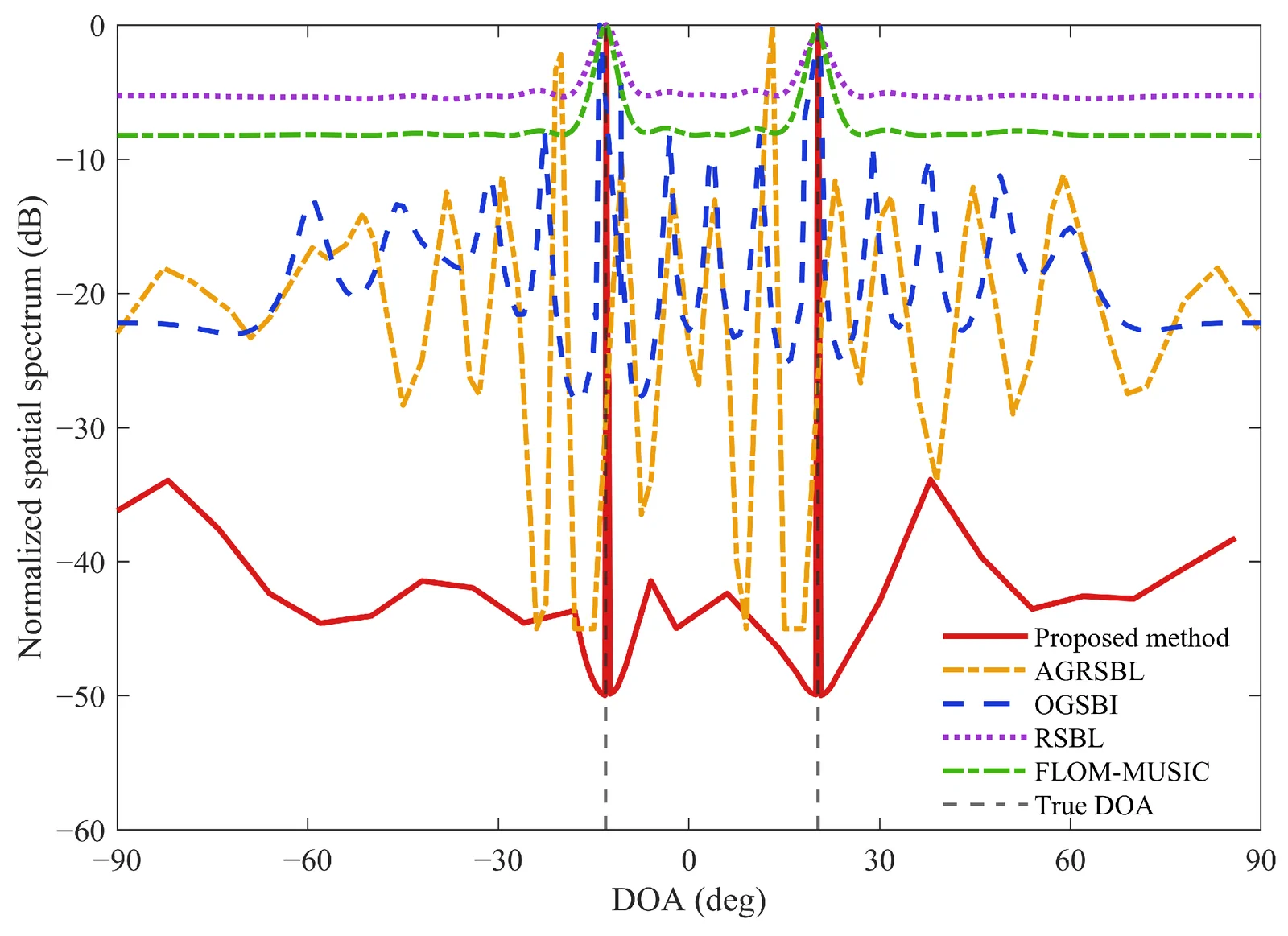
Spatial Spectrum Estimation under GMM Mixed Noise.

**Figure 3:**
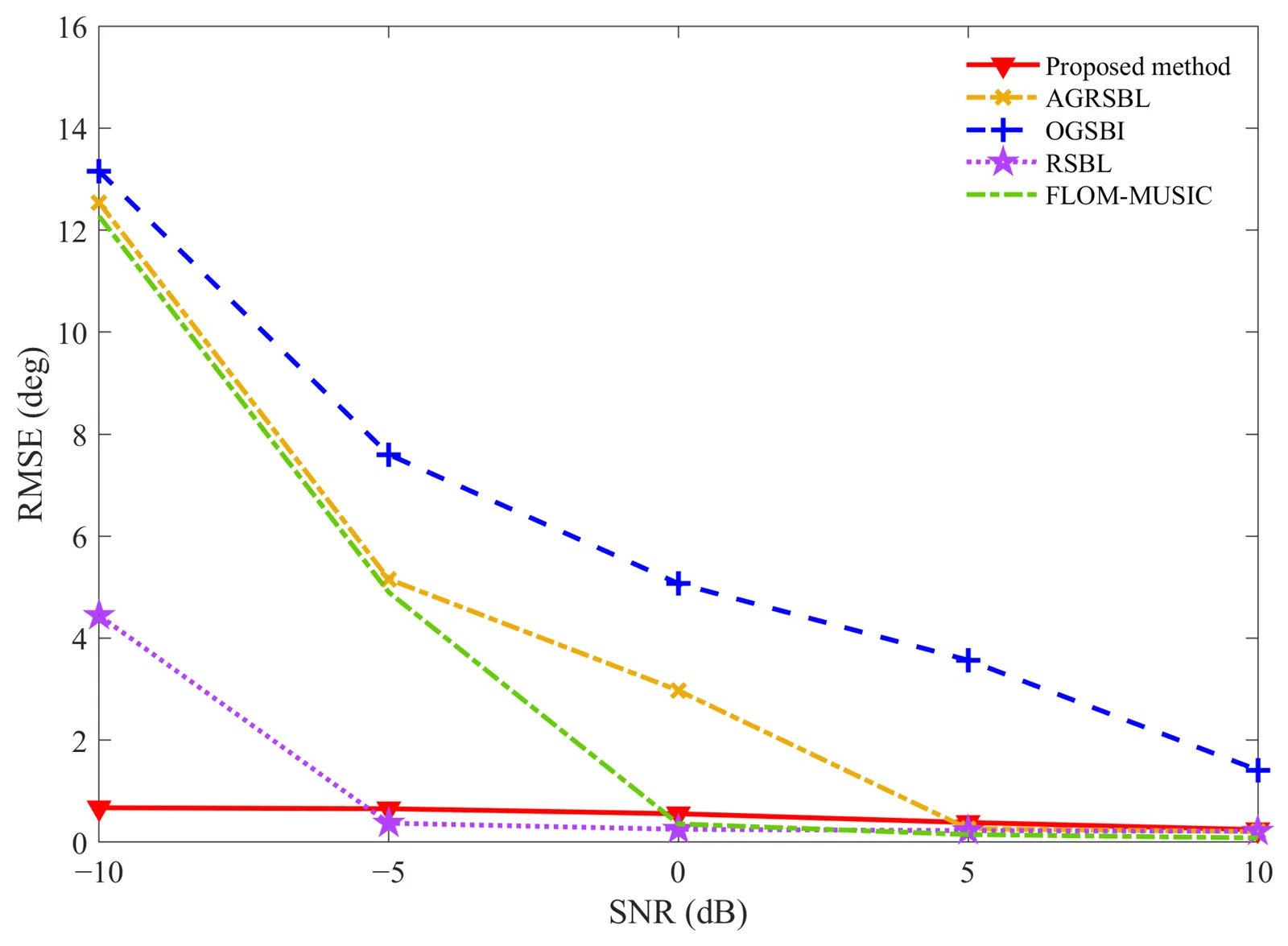
RMSE Comparison under GMM Mixed Noise.

**Figure 4:**
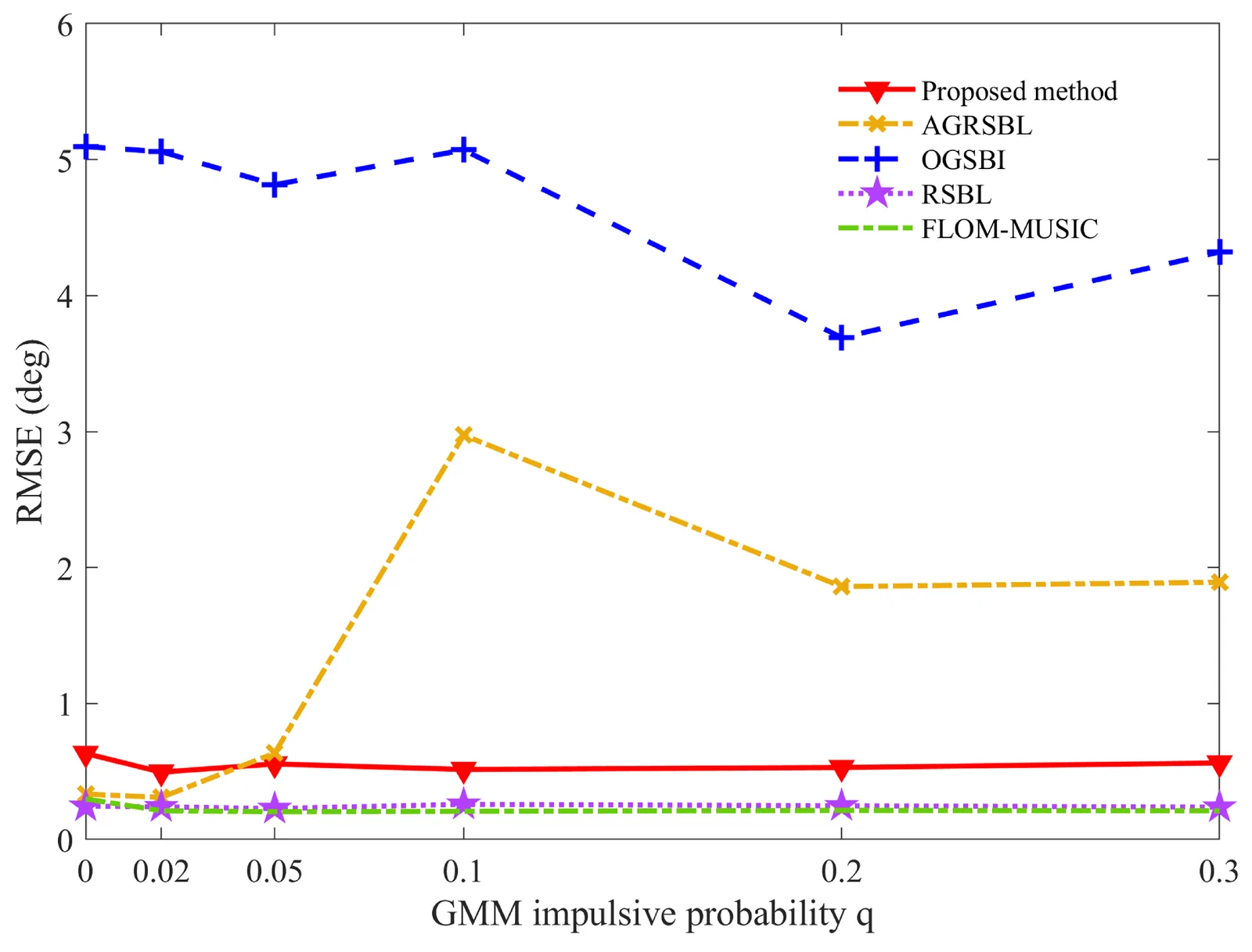
RMSE Comparison versus the GMM Impulsive Probability *q*.

**Figure 5:**
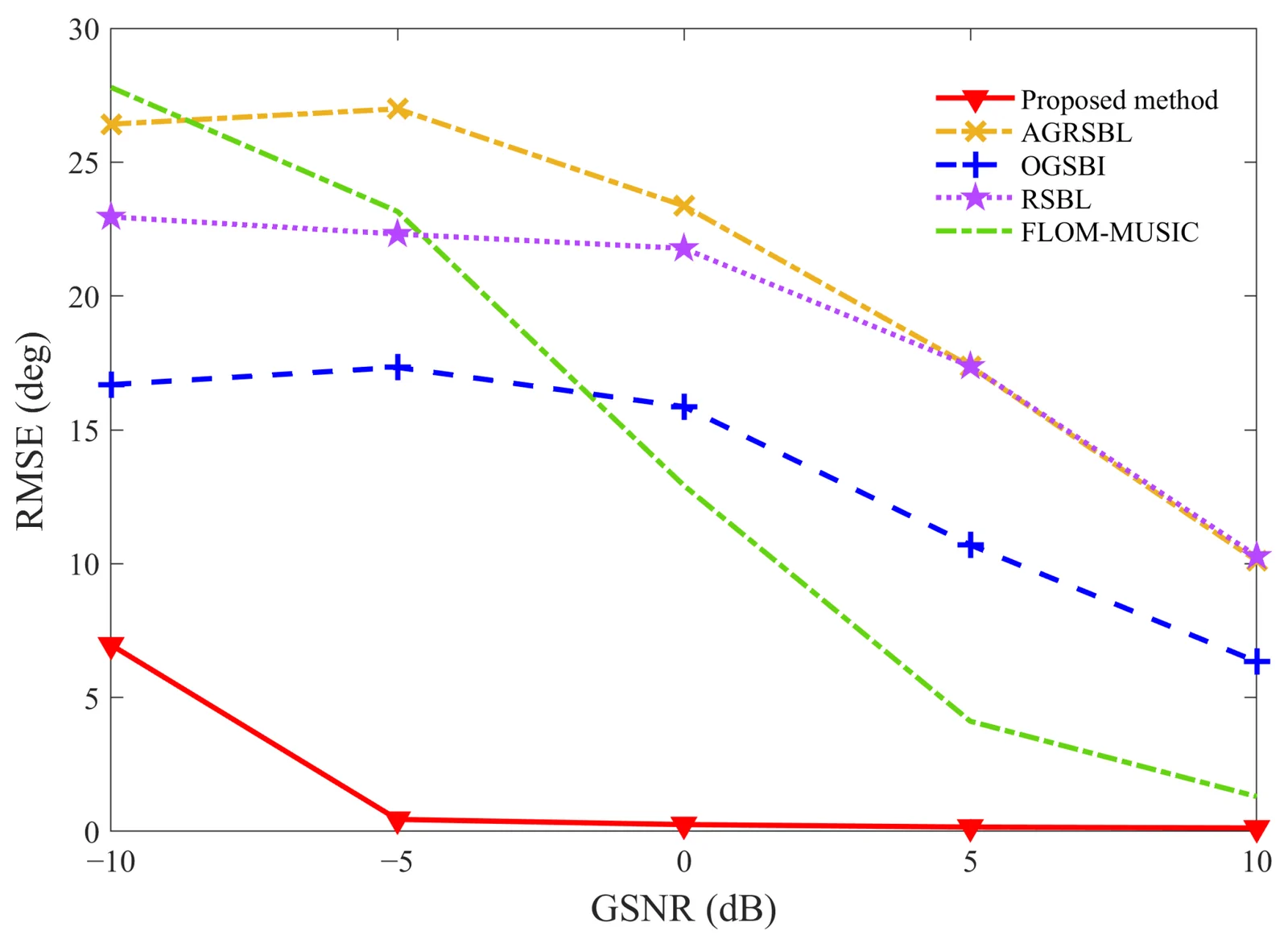
RMSE Comparison under S*α*S Mixed Noise.

**Figure 6:**
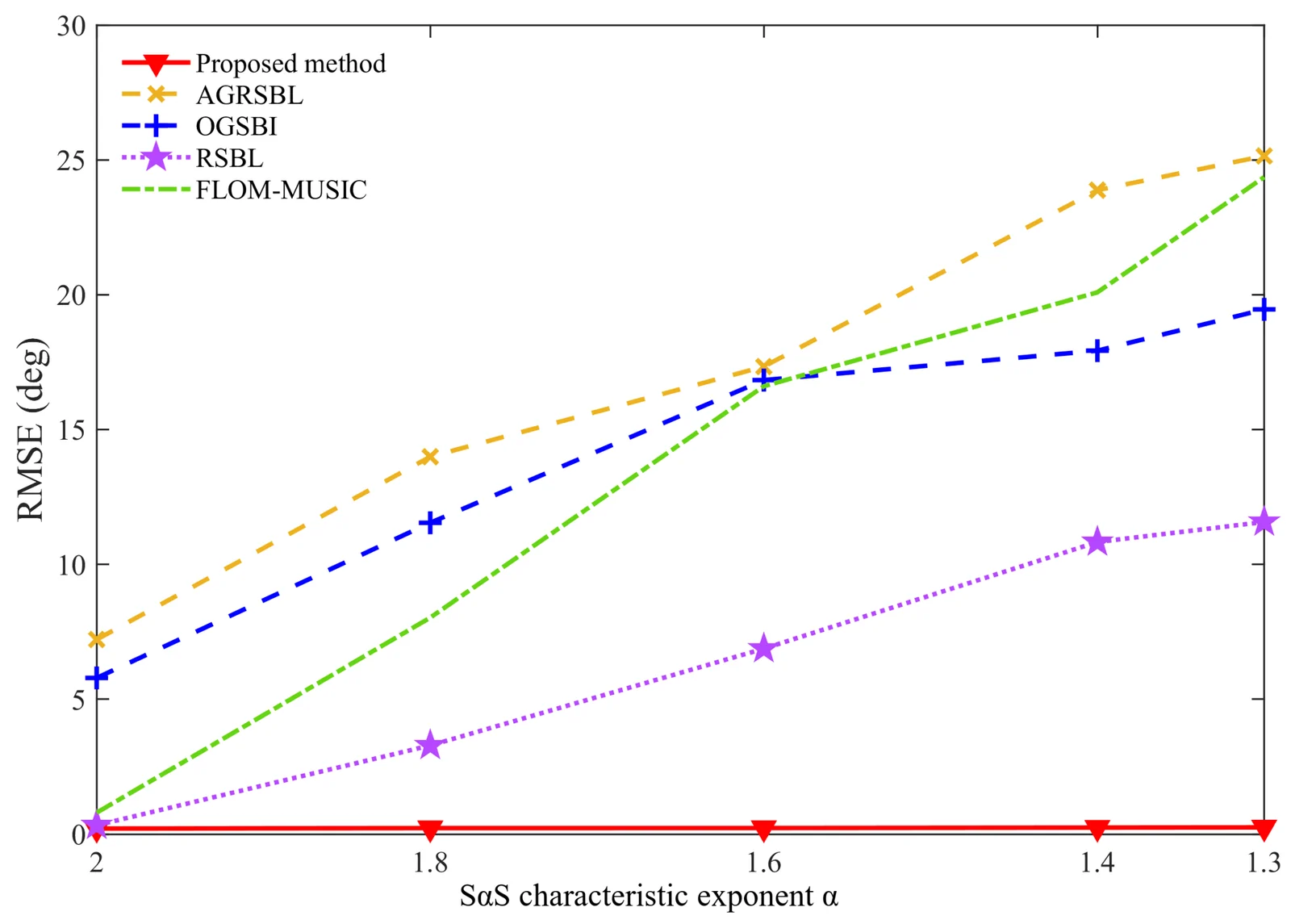
Comparison Curves of RMSE versus the S*α*S Characteristic Exponent *α*.

**Figure 7:**
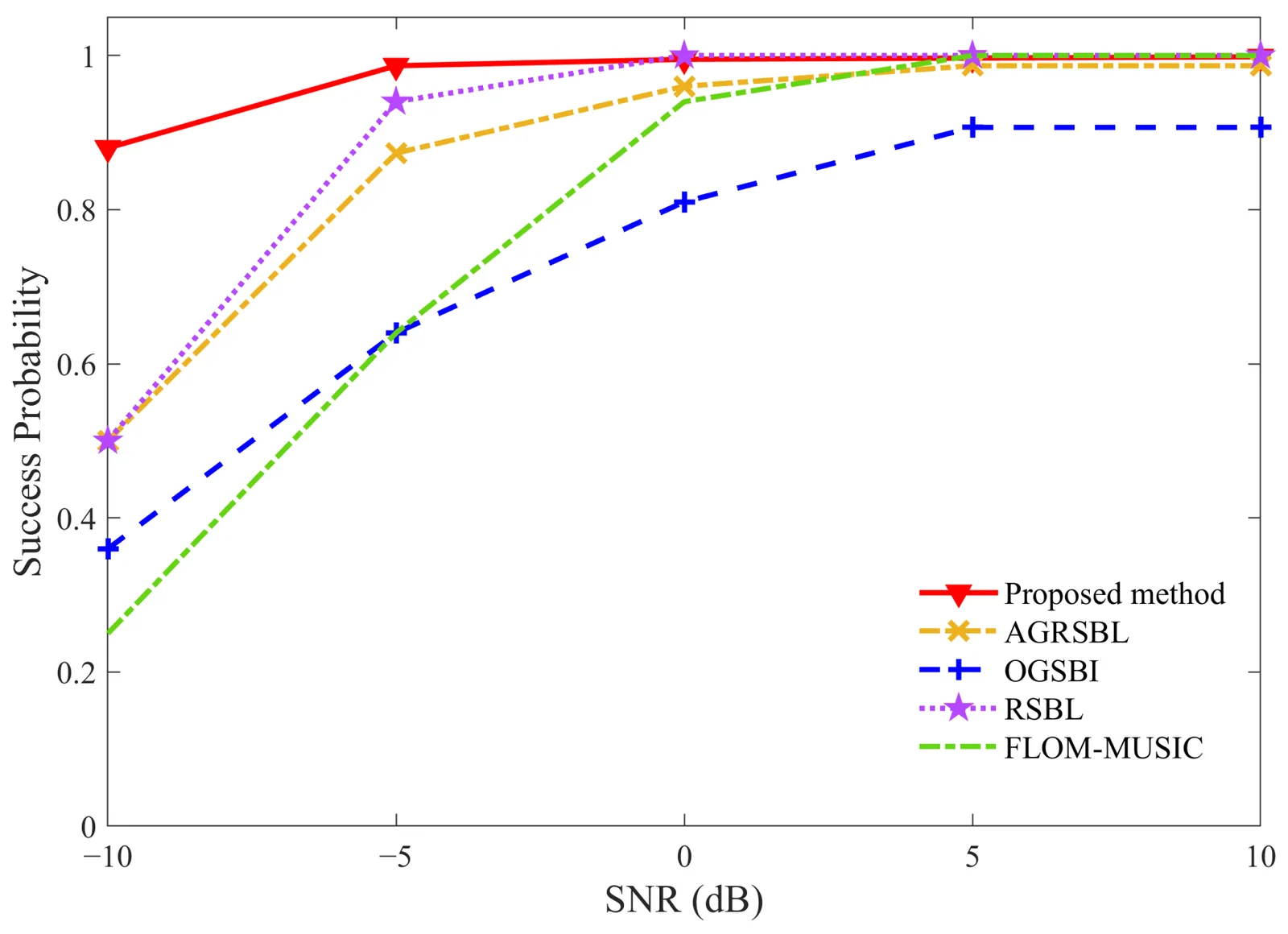
Success Probability under GMM Mixed Noise.

**Figure 8:**
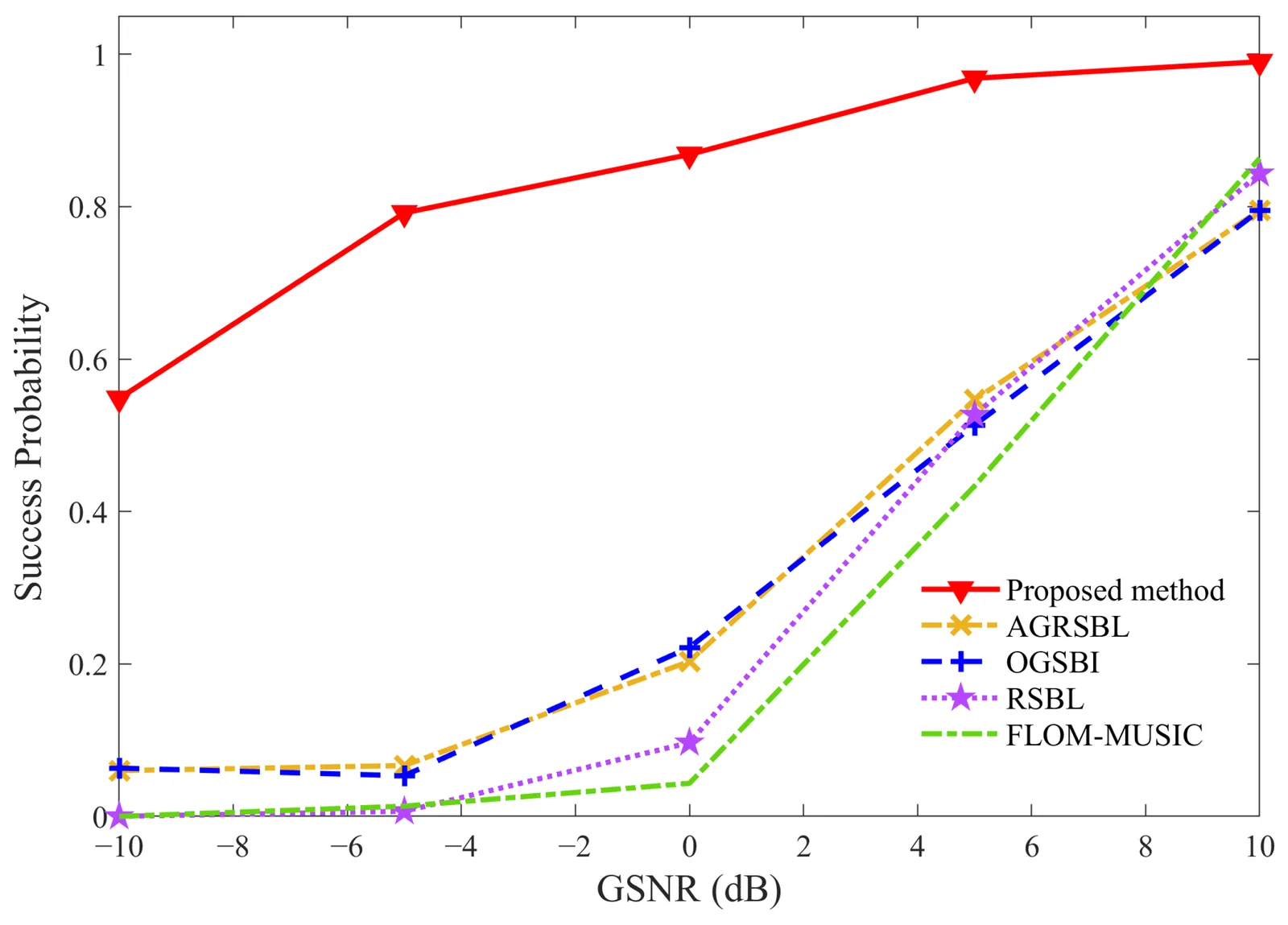
Success Probability under S*α*S Mixed Noise.

**Figure 9:**
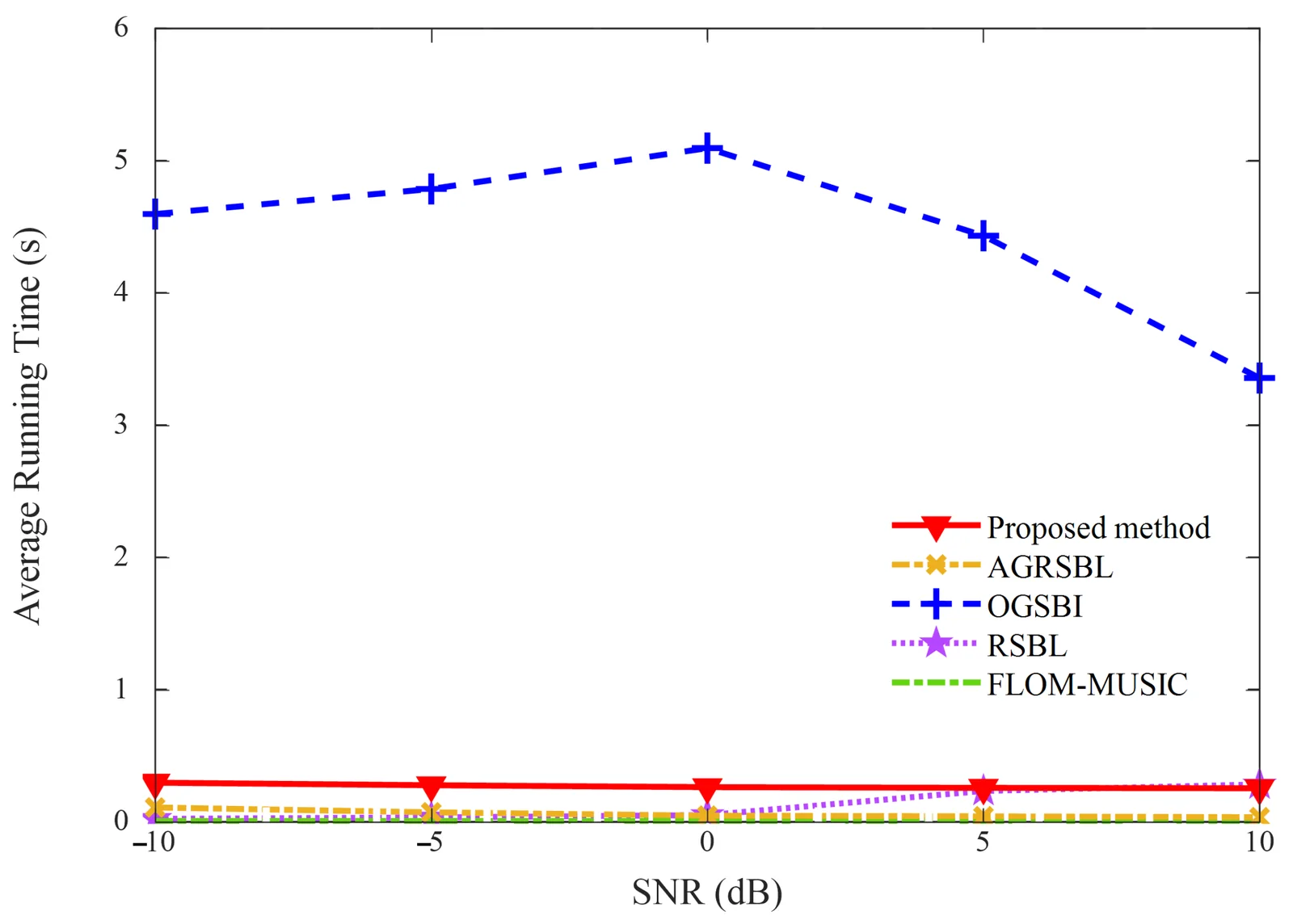
Average Running Time under GMM Mixed Noise.

**Figure 10:**
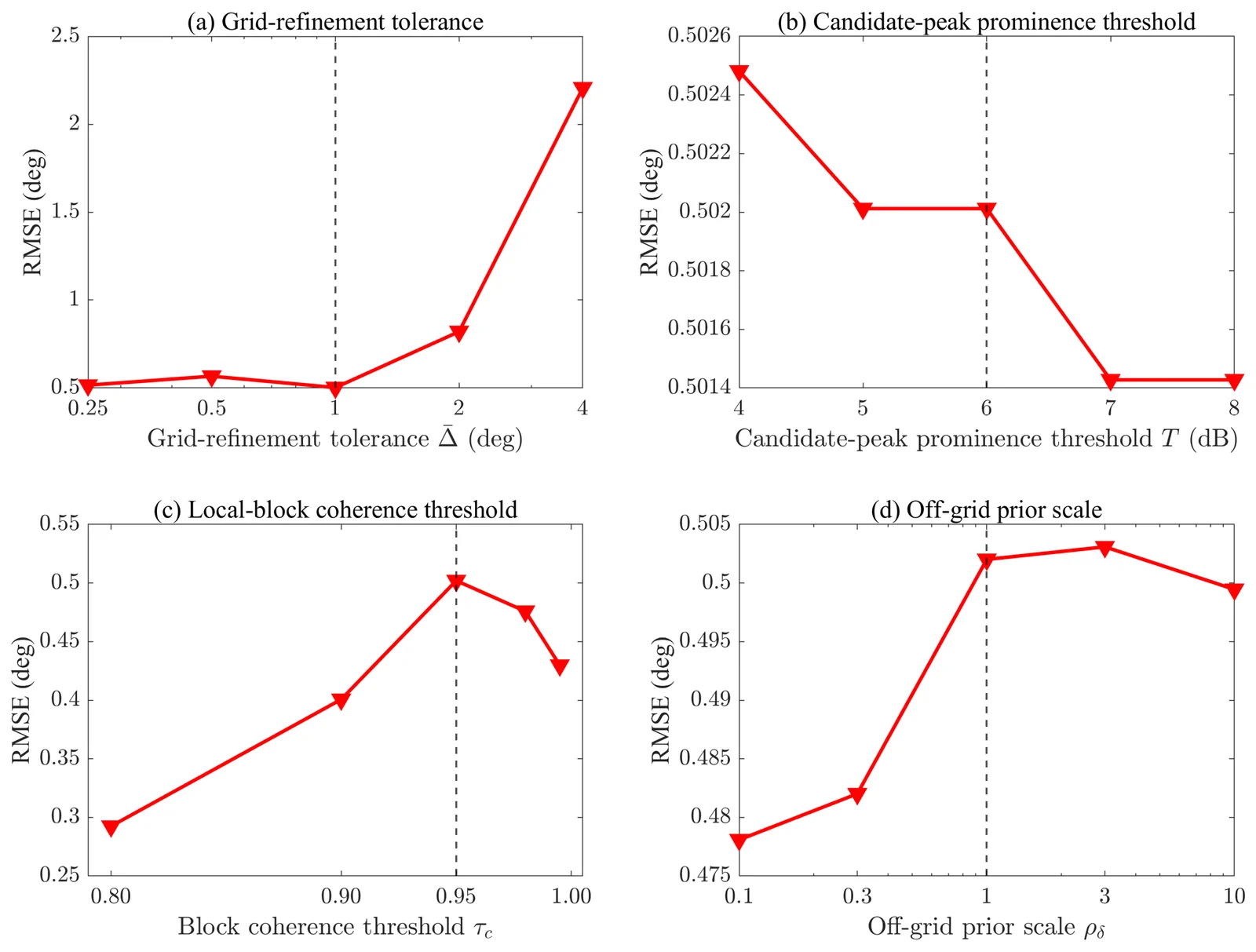
RMSE sensitivity of the proposed method to four key parameters under GMM mixed noise.

**Figure 11:**
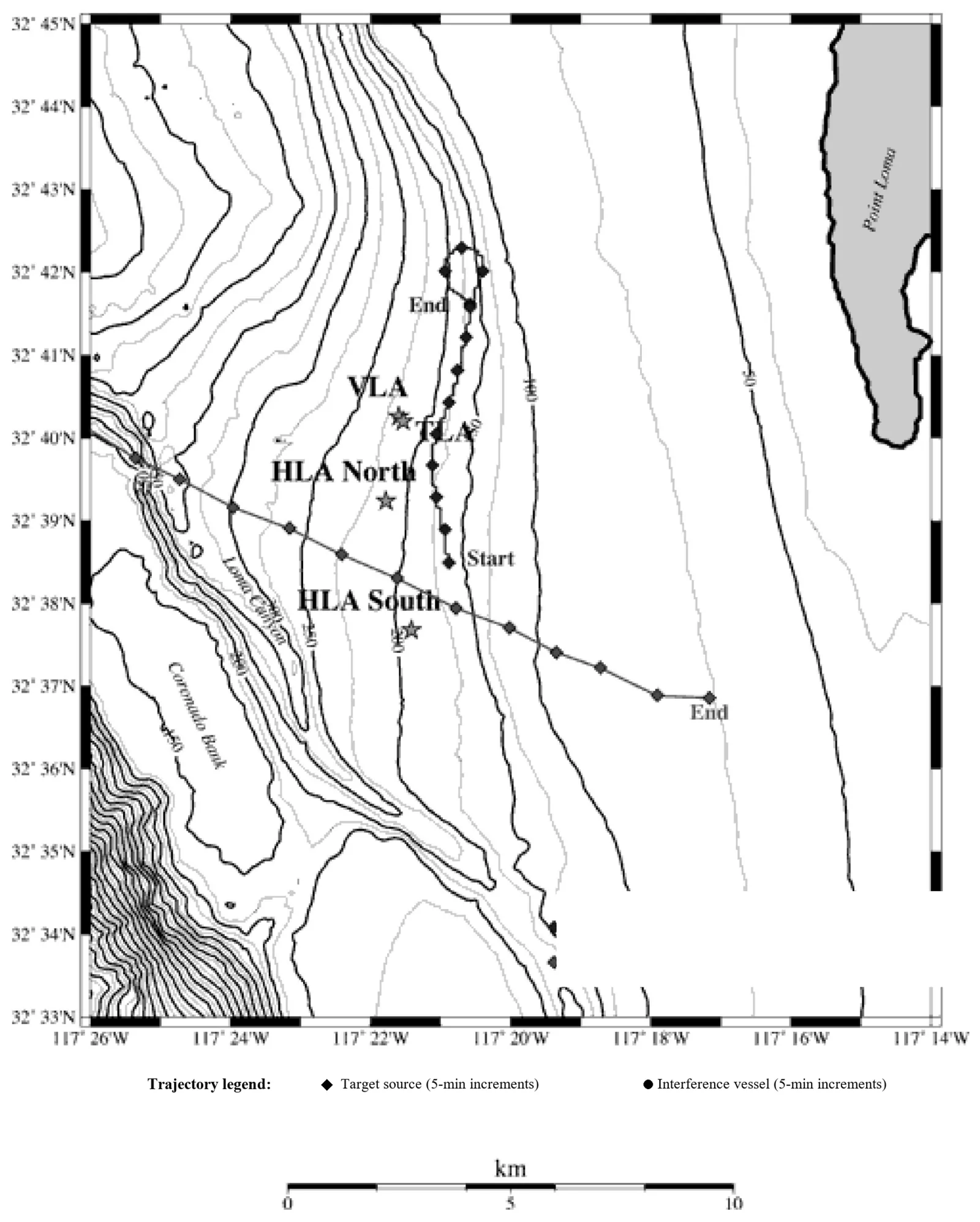
Trajectories of the SWellEx-96 experiment: Event-S59 sound source and interference. Source: reproduced from [29].

**Figure 12:**
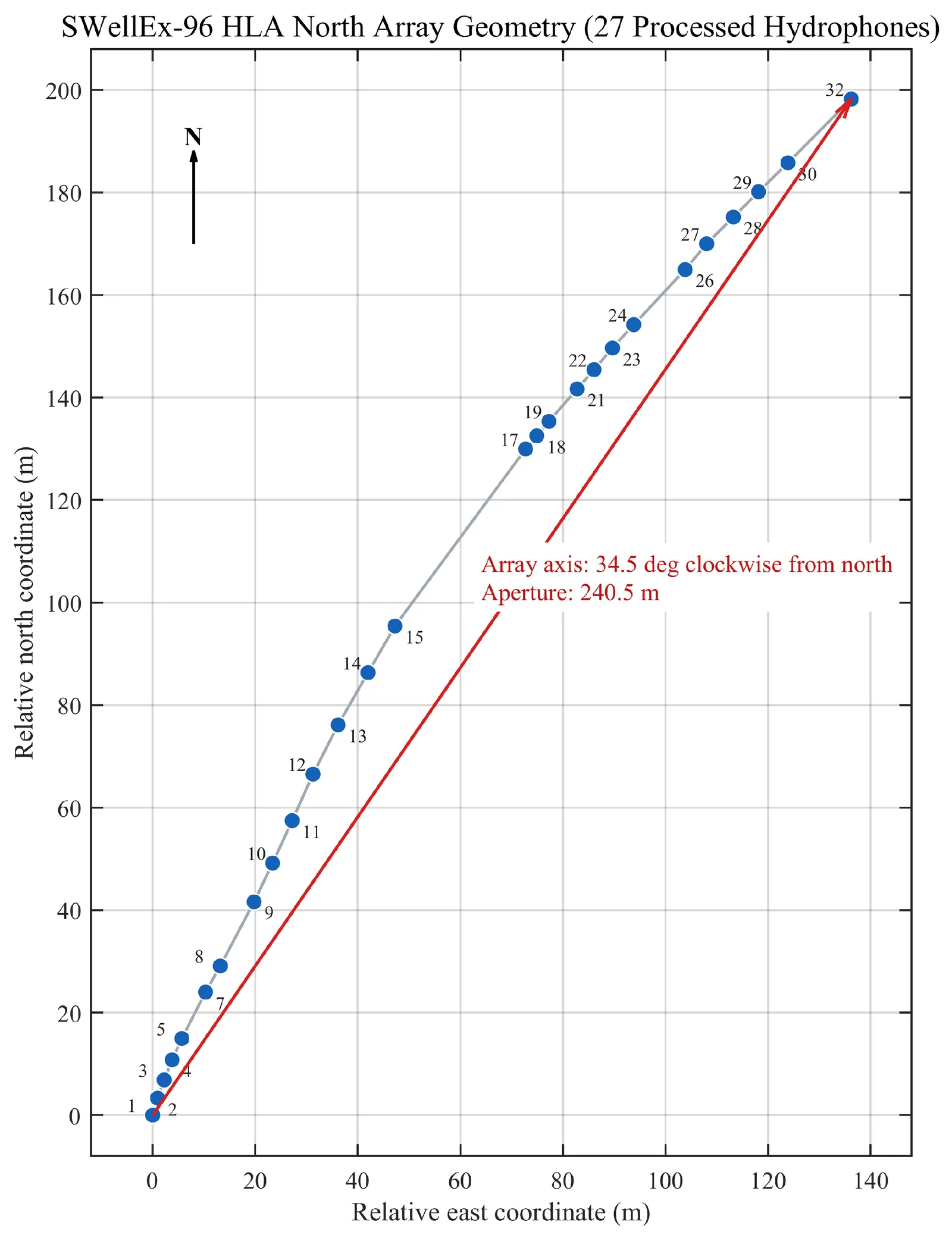
HLA North array location in the SWellEx-96 experiment.

**Figure 13:**
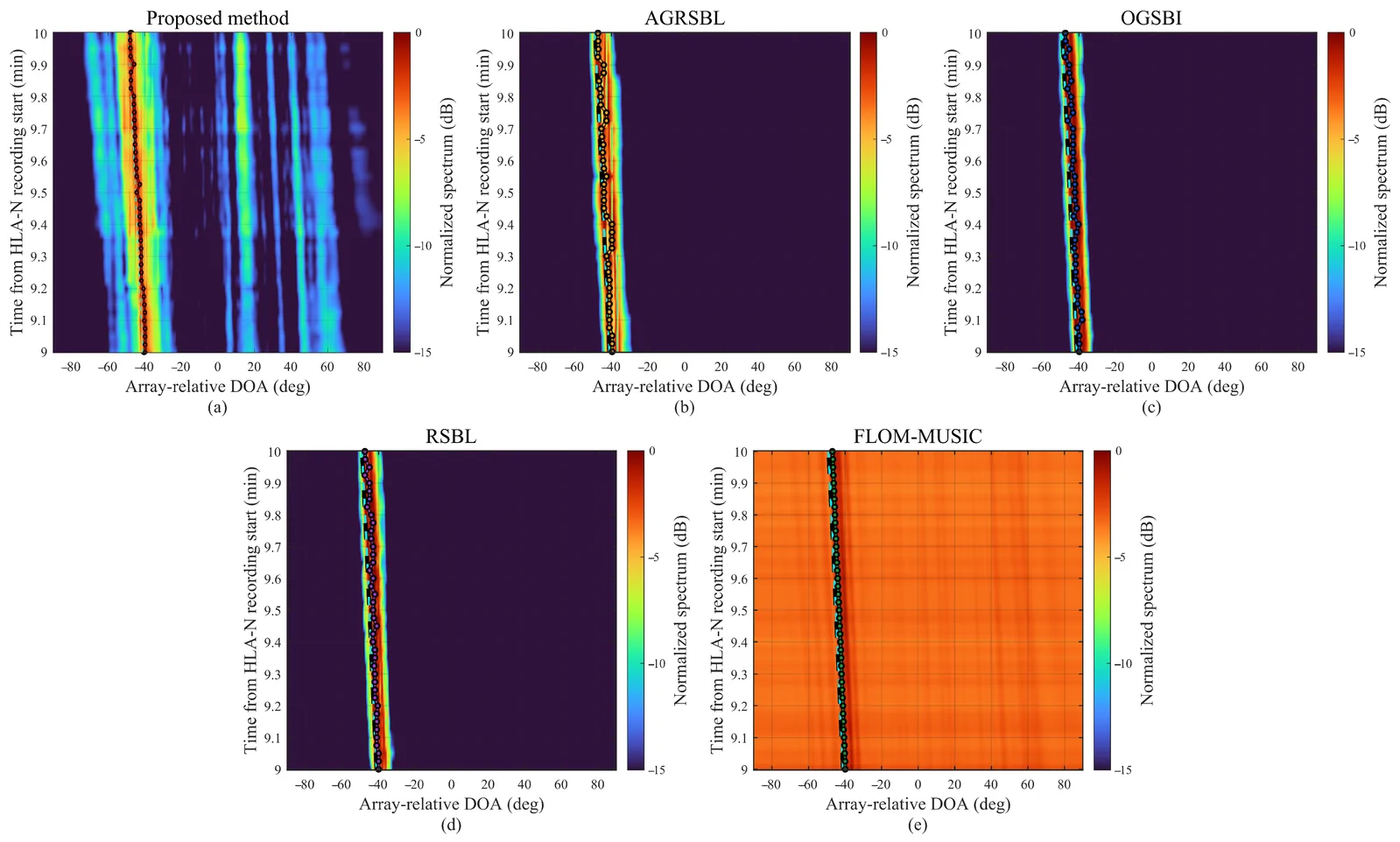
Bearing-time records of the five methods for the SWellEx-96 S59 event: (**a**) proposed method; (**b**) AGRSBL; (**c**) OGSBI; (**d**) RSBL; and (**e**) FLOM-MUSIC.

**Figure 14:**
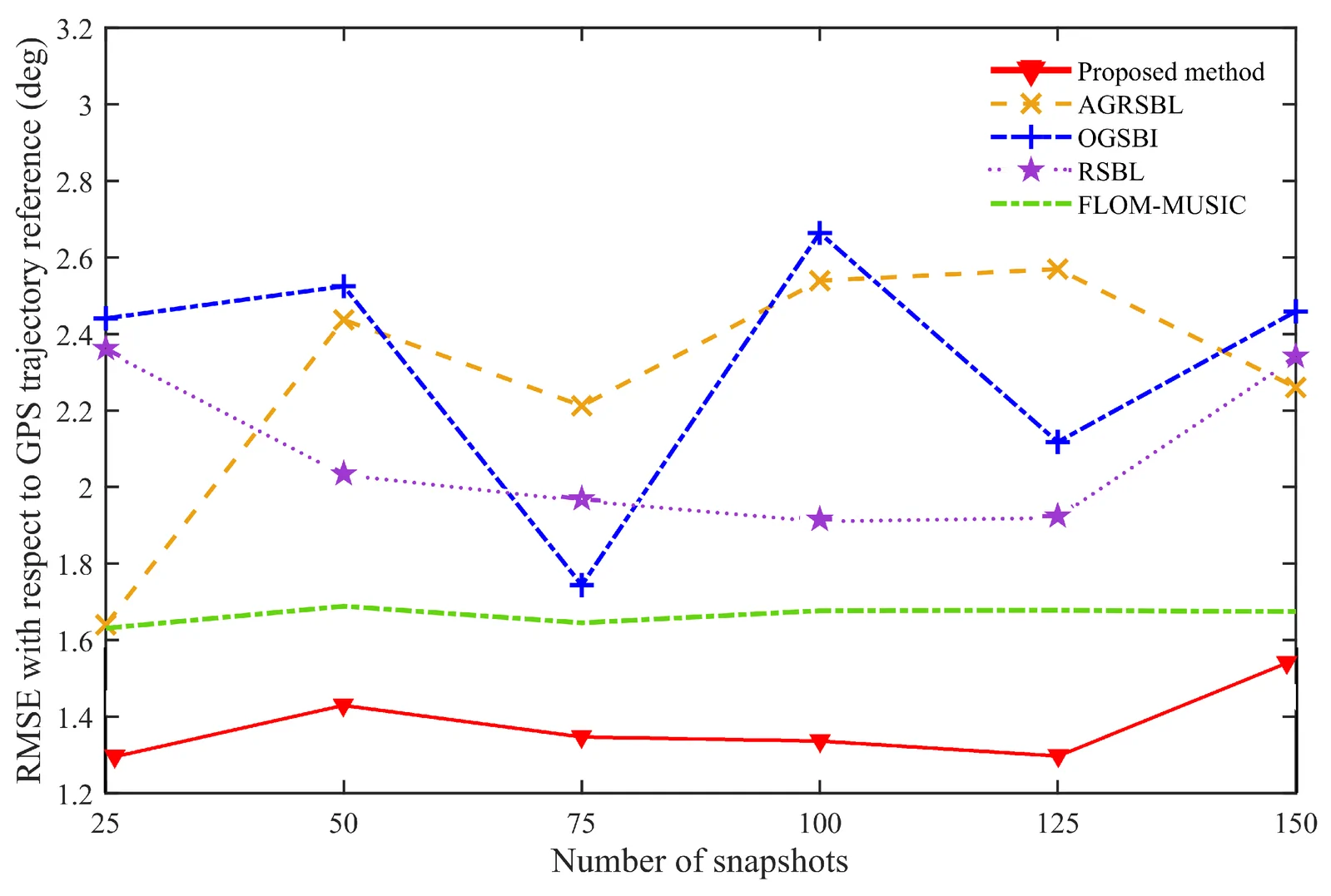
RMSE of the five methods versus the number of snapshots for the SWellEx-96 S59 event.

**Figure 15:**
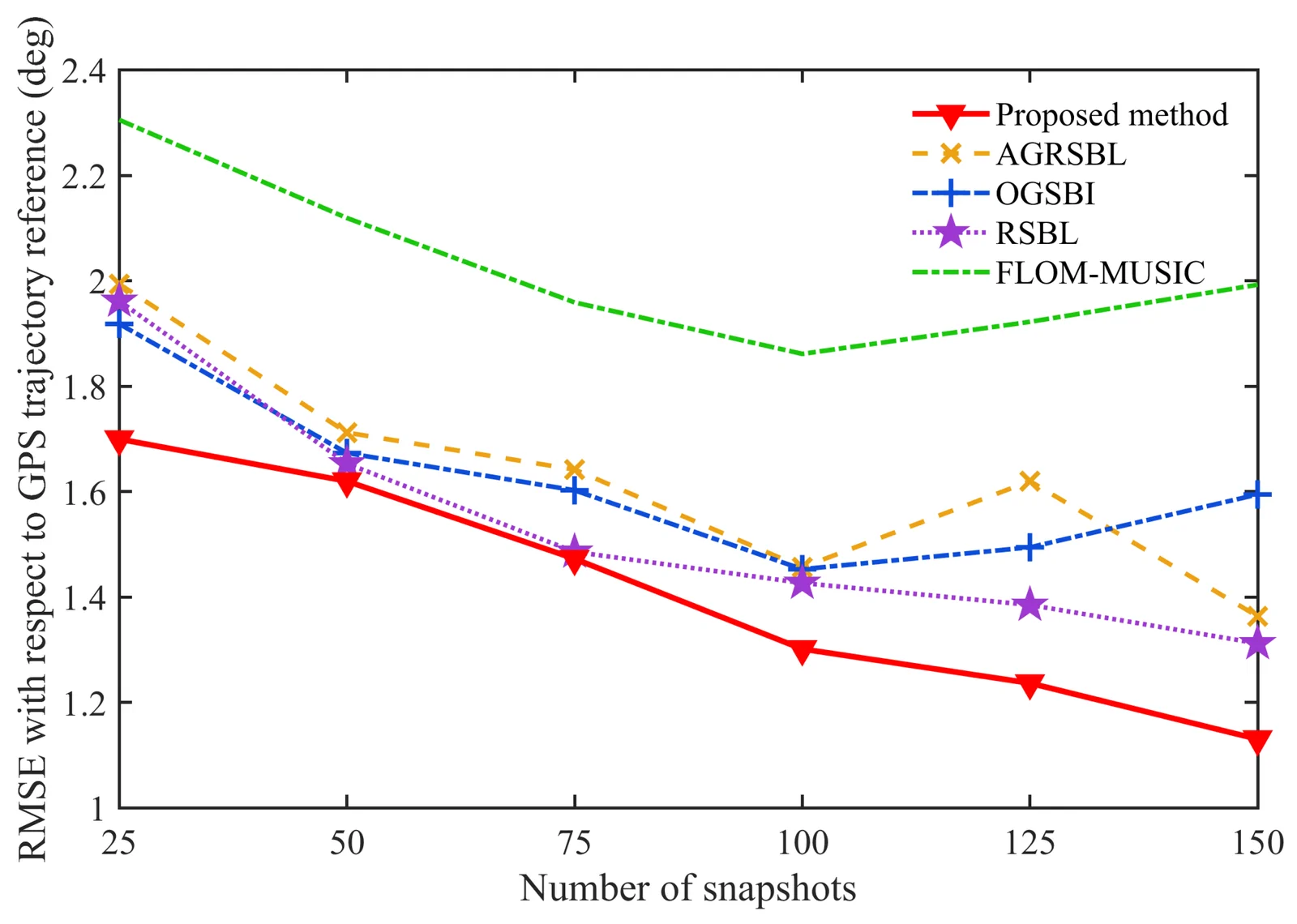
RMSE of the five methods versus the number of snapshots for the SWellEx-96 S5 event.

**Figure 16:**
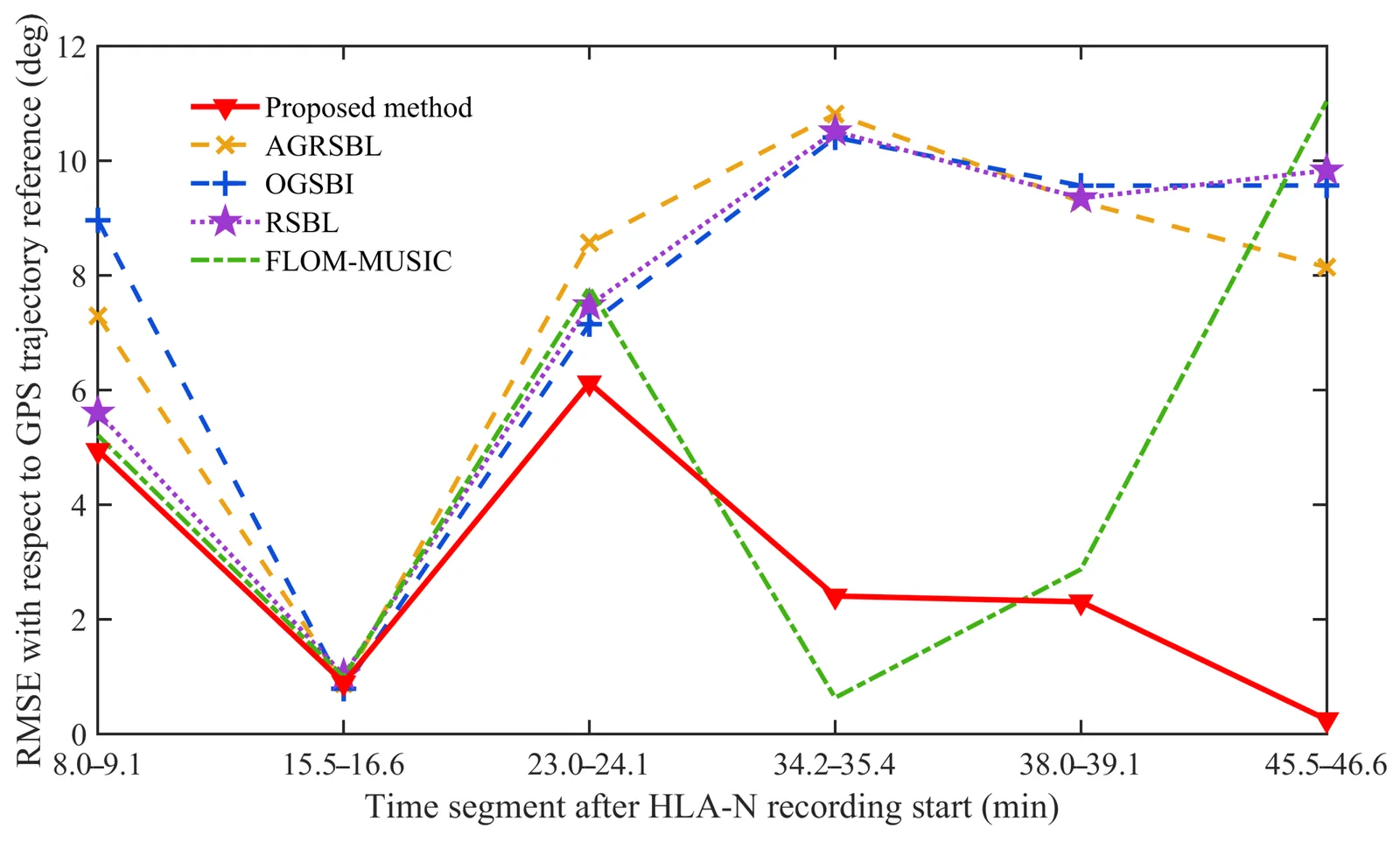
RMSE of the five methods across six time segments for the SWellEx-96 S5 event.

**Table 1:** Comparison of the proposed method with recent representative robust and off-grid SBL methods.

Method	Noise and Mismatch Setting Addressed	Main Modeling Mechanism	Posterior/Computational Design	Scope Relative to the Proposed Method
VBI for Impulsive and Nonuniform Noise [14]	Impulsive noise together with nonuniform ambient noise	Student-*t*/Bernoulli impulsive-noise modeling and nonuniform-noise representation	Variational Bayesian inference	Does not explicitly include continuous off-grid compensation or local-block covariance for coherent grids
Off-Grid SBL for MIMO Radar under Impulsive Noise [18]	Off-grid MIMO-radar DOA estimation in S*α*S impulsive noise	Impulsive-noise-aware off-grid sparse model	MIMO-radar-oriented SBL formulation	Does not explicitly model sensor-wise nonuniform noise or the identifiable temporal–spatial reliability decomposition
Off-Grid Space-Alternating SBL [21]	Off-grid DOA estimation with reduced computational cost	First-order Taylor off-grid refinement	Space-alternating SBL	Does not explicitly target joint mixed-noise modeling or preserve covariance within coherence-selected local blocks
Proposed method	Mixed marine noise and continuous grid mismatch	Temporal precision for impulsive snapshots; constrained spatial precision for degraded sensors; adaptive refinement and posterior-regularized Taylor compensation	Local-block space-alternating VBI retaining within-block covariance and avoiding full-grid covariance inversion	Jointly addresses identifiable mixed-noise weighting, local grid correlation, continuous off-grid correction, and computational tractability

**Table 2:** Mathematical symbols and their meanings.

Symbol	Representative Meaning
${\left(\cdot \right)}^{-1}$	Matrix inversion
${\left(\cdot \right)}^{T}$	Transpose
${\left(\cdot \right)}^{H}$	Conjugate transpose
${\left(\cdot \right)}^{*}$	Complex conjugate
diag(·)	Diagonal matrix
Tr(·)	Trace operation of a matrix
${∥\cdot ∥}_{2}$	$l_{2}$ norm
Re{·}	Real part of the complex value

## Data Availability

The SWellEx-96 experimental data analyzed in this study are publicly available from the Marine Physical Laboratory, Scripps Institution of Oceanography, at https://swellex96.ucsd.edu/ (accessed on 17 July 2026).
